# Supplementary material for: Role of gender in perspectives of discrimination, stigma, and attitudes relative to cervical cancer in rural Sénégal
Source: PLoS One. 2020 Apr 28;15(4):e0232291. doi: 10.1371/journal.pone.0232291 (PMC7188246; doi:10.1371/journal.pone.0232291)
Supplement: S1 File — (PDF) [file pone.0232291.s002.pdf]

2019.11.21_18Q04_K01

JAD

8/31/2019

#PREPARE Prepare RStudio and the data

##Load Libraries

```
### Load the libraries needed for this session.
library(tidyverse)
```

```
## Warning: package 'tidyverse' was built under R version 3.6.1
```

```
## -- Attaching packages ---------------------- tidyverse 1.2.1 --
```

```
## v ggplot2 3.2.1     v purrr   0.3.2
## v tibble  2.1.3     v dplyr   0.8.3
## v tidyr   1.0.0     v stringr 1.4.0
## v readr   1.3.1     v forcats 0.4.0
```

```
## Warning: package 'ggplot2' was built under R version 3.6.1
```

```
## Warning: package 'tibble' was built under R version 3.6.1
```

```
## Warning: package 'tidyr' was built under R version 3.6.1
```

```
## Warning: package 'dplyr' was built under R version 3.6.1
```

```
## -- Conflicts ------------------------- tidyverse_conflicts() --
## x dplyr::filter() masks stats::filter()
## x dplyr::lag()    masks stats::lag()
```

```r
library(here)
```

```
## here() starts at C:/Users/aldio/Google Drive/AD Scholarship _ Research/DATA ANALYSIS/18Q04 K01/18Q04 K01
```

```r
library(dplyr)
library(readr)
library(naniar)  #Replace with NA
```

```
## Warning: package 'naniar' was built under R version 3.6.1
```

```r
library(arsenal)
```

```
## Warning: package 'arsenal' was built under R version 3.6.1
```

```r
library(coin)
```

```
## Warning: package 'coin' was built under R version 3.6.1
```

```
## Loading required package: survival
```

```
## Warning: package 'survival' was built under R version 3.6.1
```

```r
library(knitr)
opts_chunk$set(tidy.opts = list(width.cutoff = 60), tidy = TRUE)
```

##Load the Data

```r
###
Q1804dta <- read_csv(here("Data", "18Q04 K01 1234Data Comb.csv"))
```

```
## Parsed with column specification:
## cols(
##   .default = col_double(),
##   ADz_SurvNum = col_character(),
```

```
##   ADz_IntvrName = col_character(),
##   ADz_SurvDate = col_character(),
##   ADz_SurvTmStrt = col_character(),
##   ADz_SurvTmEnd = col_character(),
##   ADz_PartcName = col_logical(),
##   ADz_PartcNum = col_character(),
##   SDz_AgeCat = col_logical(),
##   SDz_Ethnicity = col_logical(),
##   SDz_EthnicityOthr = col_logical(),
##   SDz_EthnicityAll = col_logical(),
##   SDz_LangPref = col_character(),
##   SDz_LangPrefAll = col_logical(),
##   BAF_CarePay = col_character(),
##   BAF_CarePayOthr = col_character(),
##   BAF_CarePayAll = col_logical(),
##   BAF_CareCost = col_character(),
##   HSz_DecTrust = col_character(),
##   HSz_Dist = col_character(),
##   HSz_ProvTyp = col_character()
##   # ... with 19 more columns
## )

## See spec(...) for full column specifications.
```

#DATA CLEANING

##Omit all N/A

```
Q1804dta <- Q1804dta %>%
 replace_with_na_all(condition = ~.x == 99) %>% #Must load library (naniar)
 replace_with_na_all(condition = ~.x == 88)

#na.omit(Q1804dta)
```

##Combine columns of categorical data to make multi-select questions complete

Combine SDLangPref and SDLangPrefOthr into new variable SDLangPrefAll and tally. Create a new variable with the combined categorical data from two columns that are each multichoice Use the function to tally the categorical data Call the function with the new variable. https://stackoverflow.com/questions/49913014/r-mutate-working-with-paste0 https://stackoverflow.cbom/questions/45696738/tallying-multiple-choice-entries-in-a-single-column-in-a-r-dataframe-programmati

Combine 2 Columns of Categorical Data. This incorporates the qualitative responses that were quantified into the existing quantified response list.

```
# SDz_LangPrefAll MUTATE COMBINE Populates SDz_LangPrefAll
# with combined Categorical Data of SDz_LangPref &
# SDz_LangPrefOthr
Q1804dta <- Q1804dta %>% mutate(SDz_LangPrefAll = paste(SDz_LangPref,
    SDz_LangPrefOthr)) %>% select(-c(SDz_LangPref, SDz_LangPrefOthr))

# Populates BAF_CarePayAll with combined Categorical Data of
# BAF_CarePay & BAF_CarePayOthr
Q1804dta <- Q1804dta %>% mutate(BAF_CarePayAll = paste(BAF_CarePay,
    BAF_CarePayOthr)) %>% select(-c(BAF_CarePay, BAF_CarePayOthr))

# Populates HSz_ProvTypAll with combined Categorical Data of
# HSz_ProvTyp & HSz_ProvTypOthr
Q1804dta <- Q1804dta %>% mutate(HSz_ProvTypAll = paste(HSz_ProvTyp,
    HSz_ProvTypOthr)) %>% select(-c(HSz_ProvTyp, HSz_ProvTypOthr))

# Populates HSz_ServLangAll with combined Categorical Data of
# HSz_ServLang & HSz_ServLangOthr
Q1804dta <- Q1804dta %>% mutate(HSz_ServLangAll = paste(HSz_ServLang,
    HSz_ServLangOthr)) %>% select(-c(HSz_ServLang, HSz_ServLangOthr))

# Populates HSz_LangInterpAll with combined Categorical Data
# of HSz_LangInterp & HSz_LangInterpOthr
Q1804dta <- Q1804dta %>% mutate(HSz_LangInterpAll = paste(HSz_LangInterp,
    HSz_LangInterpOthr)) %>% select(-c(HSz_LangInterp, HSz_LangInterpOthr))

# Populates DSz_BsisAll with combined Categorical Data of
# DSz_Bsis & DSz_BsisOthr
Q1804dta <- Q1804dta %>% mutate(DSz_BsisAll = paste(DSz_Bsis,
    DSz_BsisOthr)) %>% select(-c(DSz_Bsis, DSz_BsisOthr))

# Populates EXz_ScrnWhyAll with combined Categorical Data of
# EXz_ScrnWhy & EXz_ScrnWhyOthr
Q1804dta <- Q1804dta %>% mutate(EXz_ScrnWhyAll = paste(EXz_ScrnWhy,
    EXz_ScrnWhyOthr)) %>% select(-c(EXz_ScrnWhy, EXz_ScrnWhyOthr))

# Populates GNz_WHCDecisAll with combined Categorical Data of
```

```r
# GNz_WHCDecis & GNz_WHCDecisOthr
Q1804dta <- Q1804dta %>% mutate(GNz_WHCDecisAll = paste(GNz_WHCDecis,
    GNz_WHCDecisOthr)) %>% select(-c(GNz_WHCDecis, GNz_WHCDecisOthr))
```

##Create a new table for each multi-select variable. Use separate_rows to pull out each response Use pivot_wider to expand the table so that each variable has its own column. These will be combined with the master dataset subsequently.

```r
LangPref <- Q1804dta %>% select(ADz_PartcNum, SDz_LangPrefAll) %>%
    separate_rows(SDz_LangPrefAll) %>% mutate(LangPref_Checked = TRUE) %>%
    mutate(SDz_LangPrefAll = case_when(SDz_LangPrefAll == 1 ~
        "SDz_LangPrefAll_01", SDz_LangPrefAll == 2 ~ "SDz_LangPrefAll_02",
        SDz_LangPrefAll == 3 ~ "SDz_LangPrefAll_03", SDz_LangPrefAll ==
            4 ~ "SDz_LangPrefAll_04", SDz_LangPrefAll == 5 ~
            "SDz_LangPrefAll_05", SDz_LangPrefAll == 6 ~ "SDz_LangPrefAll_06",
        SDz_LangPrefAll == 7 ~ "SDz_LangPrefAll_07", SDz_LangPrefAll ==
            8 ~ "SDz_LangPrefAll_08", SDz_LangPrefAll == 9 ~
            "SDz_LangPrefAll_09", SDz_LangPrefAll == 10 ~ "SDz_LangPrefAll_10",
        SDz_LangPrefAll == 11 ~ "SDz_LangPrefAll_11", SDz_LangPrefAll ==
            12 ~ "SDz_LangPrefAll_12", SDz_LangPrefAll == 13 ~
            "SDz_LangPrefAll_13", SDz_LangPrefAll == 14 ~ "SDz_LangPrefAll_14",
        SDz_LangPrefAll == 15 ~ "SDz_LangPrefAll_15", SDz_LangPrefAll ==
            16 ~ "SDz_LangPrefAll_16", SDz_LangPrefAll == 17 ~
            "SDz_LangPrefAll_17", SDz_LangPrefAll == 18 ~ "SDz_LangPrefAll_18",
        SDz_LangPrefAll == 19 ~ "SDz_LangPrefAll_19", SDz_LangPrefAll ==
            20 ~ "SDz_LangPrefAll_20")) %>% pivot_wider(id_cols = ADz_PartcNum,
    names_from = SDz_LangPrefAll, values_from = LangPref_Checked,
    values_fill = list(LangPref_Checked = FALSE)) %>% select(-c("NA"))
```

```r
ServLang <- Q1804dta %>% select(ADz_PartcNum, HSz_ServLangAll) %>%
    separate_rows(HSz_ServLangAll) %>% mutate(ServLang_Checked = TRUE) %>%
    mutate(HSz_ServLangAll = case_when(HSz_ServLangAll == 1 ~
        "HSz_ServLangAll_01", HSz_ServLangAll == 2 ~ "HSz_ServLangAll_02",
        HSz_ServLangAll == 3 ~ "HSz_ServLangAll_03", HSz_ServLangAll ==
            4 ~ "HSz_ServLangAll_04", HSz_ServLangAll == 5 ~
            "HSz_ServLangAll_05", HSz_ServLangAll == 6 ~ "HSz_ServLangAll_06",
        HSz_ServLangAll == 7 ~ "HSz_ServLangAll_07", HSz_ServLangAll ==
            8 ~ "HSz_ServLangAll_08", HSz_ServLangAll == 9 ~
            "HSz_ServLangAll_09", HSz_ServLangAll == 10 ~ "HSz_ServLangAll_10",
```

```
        HSz_ServLangAll == 11 ~ "HSz_ServLangAll_11", HSz_ServLangAll ==
            12 ~ "HSz_ServLangAll_12", HSz_ServLangAll == 13 ~
            "HSz_ServLangAll_13", HSz_ServLangAll == 14 ~ "HSz_ServLangAll_14",
        HSz_ServLangAll == 15 ~ "HSz_ServLangAll_15", HSz_ServLangAll ==
            16 ~ "HSz_ServLangAll_16", HSz_ServLangAll == 17 ~
            "HSz_ServLangAll_17", HSz_ServLangAll == 18 ~ "HSz_ServLangAll_18",
        HSz_ServLangAll == 19 ~ "HSz_ServLangAll_19", HSz_ServLangAll ==
            20 ~ "HSz_ServLangAll_20", HSz_ServLangAll == 88 ~
            "88")) %>% na.omit() %>% pivot_wider(id_cols = ADz_PartcNum,
    names_from = HSz_ServLangAll, values_from = ServLang_Checked,
    values_fill = list(ServLang_Checked = FALSE)) %>% select(-c("88"))
```

```
LangInterp <- Q1804dta %>% select(ADz_PartcNum, HSz_LangInterpAll) %>%
    separate_rows(HSz_LangInterpAll) %>% mutate(LangInterp_Checked = TRUE) %>%
    mutate(HSz_LangInterpAll = case_when(HSz_LangInterpAll ==
        1 ~ "HSz_LangInterpAll_01", HSz_LangInterpAll == 2 ~
        "HSz_LangInterpAll_02", HSz_LangInterpAll == 3 ~ "HSz_LangInterpAll_03",
        HSz_LangInterpAll == 4 ~ "HSz_LangInterpAll_04", HSz_LangInterpAll ==
            5 ~ "HSz_LangInterpAll_05", HSz_LangInterpAll ==
            6 ~ "HSz_LangInterpAll_06", HSz_LangInterpAll ==
            7 ~ "HSz_LangInterpAll_07", HSz_LangInterpAll ==
            8 ~ "HSz_LangInterpAll_08", HSz_LangInterpAll ==
            9 ~ "HSz_LangInterpAll_09", HSz_LangInterpAll ==
            10 ~ "HSz_LangInterpAll_10", HSz_LangInterpAll ==
            11 ~ "HSz_LangInterpAll_11", HSz_LangInterpAll ==
            12 ~ "HSz_LangInterpAll_12", HSz_LangInterpAll ==
            13 ~ "HSz_LangInterpAll_13", HSz_LangInterpAll ==
            14 ~ "HSz_LangInterpAll_14", HSz_LangInterpAll ==
            15 ~ "HSz_LangInterpAll_15", HSz_LangInterpAll ==
            16 ~ "HSz_LangInterpAll_16", HSz_LangInterpAll ==
            17 ~ "HSz_LangInterpAll_17", HSz_LangInterpAll ==
            18 ~ "HSz_LangInterpAll_18", HSz_LangInterpAll ==
            19 ~ "HSz_LangInterpAll_19", HSz_LangInterpAll ==
            20 ~ "HSz_LangInterpAll_20", HSz_LangInterpAll ==
            88 ~ "88", HSz_LangInterpAll == 99 ~ "99")) %>% na.omit() %>%
    pivot_wider(id_cols = ADz_PartcNum, names_from = HSz_LangInterpAll,
        values_from = LangInterp_Checked, values_fill = list(LangInterp_Checked = FALSE)) %>%
    select(-c("88"))
```

```
CarePay <- Q1804dta %>% select(ADz_PartcNum, BAF_CarePayAll) %>%
    separate_rows(BAF_CarePayAll) %>% mutate(CarePay_Checked = TRUE) %>%
    mutate(BAF_CarePayAll = case_when(BAF_CarePayAll == 1 ~ "BAF_CarePayAll_01",
        BAF_CarePayAll == 2 ~ "BAF_CarePayAll_02", BAF_CarePayAll ==
            3 ~ "BAF_CarePayAll_03", BAF_CarePayAll == 4 ~ "BAF_CarePayAll_04",
        BAF_CarePayAll == 5 ~ "BAF_CarePayAll_05", BAF_CarePayAll ==
            6 ~ "BAF_CarePayAll_06", BAF_CarePayAll == 7 ~ "BAF_CarePayAll_07",
        BAF_CarePayAll == 8 ~ "BAF_CarePayAll_08", BAF_CarePayAll ==
            9 ~ "BAF_CarePayAll_09", BAF_CarePayAll == 10 ~ "BAF_CarePayAll_10",
        BAF_CarePayAll == 11 ~ "BAF_CarePayAll_11", BAF_CarePayAll ==
            12 ~ "BAF_CarePayAll_12", BAF_CarePayAll == 13 ~
            "BAF_CarePayAll_13", BAF_CarePayAll == 14 ~ "BAF_CarePayAll_14",
        BAF_CarePayAll == 15 ~ "BAF_CarePayAll_15", BAF_CarePayAll ==
            16 ~ "BAF_CarePayAll_16", BAF_CarePayAll == 17 ~
            "BAF_CarePayAll_17", BAF_CarePayAll == 18 ~ "BAF_CarePayAll_18",
        BAF_CarePayAll == 19 ~ "BAF_CarePayAll_19", BAF_CarePayAll ==
            20 ~ "BAF_CarePayAll_20", BAF_CarePayAll == 88 ~
            "88")) %>% na.omit() %>% pivot_wider(id_cols = ADz_PartcNum,
    names_from = BAF_CarePayAll, values_from = CarePay_Checked,
    values_fill = list(CarePay_Checked = FALSE)) %>% select(-c("88"))
```

```
ProvTyp <- Q1804dta %>% select(ADz_PartcNum, HSz_ProvTypAll) %>%
    separate_rows(HSz_ProvTypAll) %>% mutate(ProvTyp_Checked = TRUE) %>%
    mutate(HSz_ProvTypAll = case_when(HSz_ProvTypAll == 1 ~ "HSz_ProvTypAll_01",
        HSz_ProvTypAll == 2 ~ "HSz_ProvTypAll_02", HSz_ProvTypAll ==
            3 ~ "HSz_ProvTypAll_03", HSz_ProvTypAll == 4 ~ "HSz_ProvTypAll_04",
        HSz_ProvTypAll == 5 ~ "HSz_ProvTypAll_05", HSz_ProvTypAll ==
            6 ~ "HSz_ProvTypAll_06", HSz_ProvTypAll == 7 ~ "HSz_ProvTypAll_07",
        HSz_ProvTypAll == 8 ~ "HSz_ProvTypAll_08", HSz_ProvTypAll ==
            9 ~ "HSz_ProvTypAll_09", HSz_ProvTypAll == 10 ~ "HSz_ProvTypAll_10",
        HSz_ProvTypAll == 11 ~ "HSz_ProvTypAll_11", HSz_ProvTypAll ==
            12 ~ "HSz_ProvTypAll_12", HSz_ProvTypAll == 13 ~
            "HSz_ProvTypAll_13", HSz_ProvTypAll == 14 ~ "HSz_ProvTypAll_14",
        HSz_ProvTypAll == 15 ~ "HSz_ProvTypAll_15", HSz_ProvTypAll ==
            16 ~ "HSz_ProvTypAll_16", HSz_ProvTypAll == 17 ~
            "HSz_ProvTypAll_17", HSz_ProvTypAll == 18 ~ "HSz_ProvTypAll_18",
        HSz_ProvTypAll == 19 ~ "HSz_ProvTypAll_19", HSz_ProvTypAll ==
            20 ~ "HSz_ProvTypAll_20")) %>% na.omit() %>% pivot_wider(id_cols = ADz_PartcNum,
```

```
    names_from = HSz_ProvTypAll, values_from = ProvTyp_Checked,
    values_fill = list(ProvTyp_Checked = FALSE))
```

```
Bsis <- Q1804dta %>% select(ADz_PartcNum, DSz_BsisAll) %>% separate_rows(DSz_BsisAll) %>%
    mutate(Bsis_Checked = TRUE) %>% mutate(DSz_BsisAll = case_when(DSz_BsisAll ==
    1 ~ "DSz_BsisAll_01", DSz_BsisAll == 2 ~ "DSz_BsisAll_02",
    DSz_BsisAll == 3 ~ "DSz_BsisAll_03", DSz_BsisAll == 4 ~ "DSz_BsisAll_04",
    DSz_BsisAll == 5 ~ "DSz_BsisAll_05", DSz_BsisAll == 6 ~ "DSz_BsisAll_06",
    DSz_BsisAll == 7 ~ "DSz_BsisAll_07", DSz_BsisAll == 8 ~ "DSz_BsisAll_08",
    DSz_BsisAll == 9 ~ "DSz_BsisAll_09", DSz_BsisAll == 10 ~
        "DSz_BsisAll_10", DSz_BsisAll == 11 ~ "DSz_BsisAll_11",
    DSz_BsisAll == 12 ~ "DSz_BsisAll_12", DSz_BsisAll == 13 ~
        "DSz_BsisAll_13", DSz_BsisAll == 14 ~ "DSz_BsisAll_14",
    DSz_BsisAll == 15 ~ "DSz_BsisAll_15", DSz_BsisAll == 16 ~
        "DSz_BsisAll_16", DSz_BsisAll == 17 ~ "DSz_BsisAll_17",
    DSz_BsisAll == 18 ~ "DSz_BsisAll_18", DSz_BsisAll == 19 ~
        "DSz_BsisAll_19", DSz_BsisAll == 20 ~ "DSz_BsisAll_20",
    DSz_BsisAll == 88 ~ "88", DSz_BsisAll == 99 ~ "99")) %>%
    na.omit() %>% pivot_wider(id_cols = ADz_PartcNum, names_from = DSz_BsisAll,
    values_from = Bsis_Checked, values_fill = list(Bsis_Checked = FALSE)) %>%
    select(-c("88"))
```

```
ScrnWhy <- Q1804dta %>% select(ADz_PartcNum, EXz_ScrnWhyAll) %>%
    separate_rows(EXz_ScrnWhyAll) %>% mutate(ScrnWhy_Checked = TRUE) %>%
    mutate(EXz_ScrnWhyAll = case_when(EXz_ScrnWhyAll == 1 ~ "EXz_ScrnWhyAll_01",
        EXz_ScrnWhyAll == 2 ~ "EXz_ScrnWhyAll_02", EXz_ScrnWhyAll ==
            3 ~ "EXz_ScrnWhyAll_03", EXz_ScrnWhyAll == 4 ~ "EXz_ScrnWhyAll_04",
        EXz_ScrnWhyAll == 5 ~ "EXz_ScrnWhyAll_05", EXz_ScrnWhyAll ==
            6 ~ "EXz_ScrnWhyAll_06", EXz_ScrnWhyAll == 7 ~ "EXz_ScrnWhyAll_07",
        EXz_ScrnWhyAll == 8 ~ "EXz_ScrnWhyAll_08", EXz_ScrnWhyAll ==
            9 ~ "EXz_ScrnWhyAll_09", EXz_ScrnWhyAll == 10 ~ "EXz_ScrnWhyAll_10",
        EXz_ScrnWhyAll == 11 ~ "EXz_ScrnWhyAll_11", EXz_ScrnWhyAll ==
            12 ~ "EXz_ScrnWhyAll_12", EXz_ScrnWhyAll == 13 ~
            "EXz_ScrnWhyAll_13", EXz_ScrnWhyAll == 14 ~ "EXz_ScrnWhyAll_14",
        EXz_ScrnWhyAll == 15 ~ "EXz_ScrnWhyAll_15", EXz_ScrnWhyAll ==
            16 ~ "EXz_ScrnWhyAll_16", EXz_ScrnWhyAll == 17 ~
            "EXz_ScrnWhyAll_17", EXz_ScrnWhyAll == 18 ~ "EXz_ScrnWhyAll_18",
        EXz_ScrnWhyAll == 19 ~ "EXz_ScrnWhyAll_19", EXz_ScrnWhyAll ==
```

```
        20 ~ "EXz_ScrnWhyAll_20")) %>% na.omit() %>% pivot_wider(id_cols = ADz_PartcNum,
    names_from = EXz_ScrnWhyAll, values_from = ScrnWhy_Checked,
    values_fill = list(ScrnWhy_Checked = FALSE))
```

```
WHCDecis <- Q1804dta %>% select(ADz_PartcNum, GNz_WHCDecisAll) %>%
    separate_rows(GNz_WHCDecisAll) %>% mutate(WHCDecis_Checked = TRUE) %>%
    mutate(GNz_WHCDecisAll = case_when(GNz_WHCDecisAll == 1 ~
        "GNz_WHCDecisAll_01", GNz_WHCDecisAll == 2 ~ "GNz_WHCDecisAll_02",
        GNz_WHCDecisAll == 3 ~ "GNz_WHCDecisAll_03", GNz_WHCDecisAll ==
            4 ~ "GNz_WHCDecisAll_04", GNz_WHCDecisAll == 5 ~
            "GNz_WHCDecisAll_05", GNz_WHCDecisAll == 6 ~ "GNz_WHCDecisAll_06",
        GNz_WHCDecisAll == 7 ~ "GNz_WHCDecisAll_07", GNz_WHCDecisAll ==
            8 ~ "GNz_WHCDecisAll_08", GNz_WHCDecisAll == 9 ~
            "GNz_WHCDecisAll_09", GNz_WHCDecisAll == 10 ~ "GNz_WHCDecisAll_10",
        GNz_WHCDecisAll == 11 ~ "GNz_WHCDecisAll_11", GNz_WHCDecisAll ==
            12 ~ "GNz_WHCDecisAll_12", GNz_WHCDecisAll == 13 ~
            "GNz_WHCDecisAll_13", GNz_WHCDecisAll == 14 ~ "GNz_WHCDecisAll_14",
        GNz_WHCDecisAll == 15 ~ "GNz_WHCDecisAll_15", GNz_WHCDecisAll ==
            16 ~ "GNz_WHCDecisAll_16", GNz_WHCDecisAll == 17 ~
            "GNz_WHCDecisAll_17", GNz_WHCDecisAll == 18 ~ "GNz_WHCDecisAll_18",
        GNz_WHCDecisAll == 19 ~ "GNz_WHCDecisAll_19", GNz_WHCDecisAll ==
            20 ~ "GNz_WHCDecisAll_20", GNz_WHCDecisAll == 88 ~
            "88", GNz_WHCDecisAll == 99 ~ "99")) %>% na.omit() %>%
    pivot_wider(id_cols = ADz_PartcNum, names_from = GNz_WHCDecisAll,
        values_from = WHCDecis_Checked, values_fill = list(WHCDecis_Checked = FALSE))
```

```
DecTrust <- Q1804dta %>% select(ADz_PartcNum, HSz_DecTrust) %>%
    separate_rows(HSz_DecTrust) %>% mutate(DecTrust_Checked = TRUE) %>%
    mutate(HSz_DecTrust = case_when(HSz_DecTrust == 1 ~ "HSz_DecTrust_01",
        HSz_DecTrust == 2 ~ "HSz_DecTrust_02", HSz_DecTrust ==
            3 ~ "HSz_DecTrust_03", HSz_DecTrust == 4 ~ "HSz_DecTrust_04",
        HSz_DecTrust == 5 ~ "HSz_DecTrust_05", HSz_DecTrust ==
            6 ~ "HSz_DecTrust_06", HSz_DecTrust == 7 ~ "HSz_DecTrust_07",
        HSz_DecTrust == 8 ~ "HSz_DecTrust_08", HSz_DecTrust ==
            9 ~ "HSz_DecTrust_09", HSz_DecTrust == 10 ~ "HSz_DecTrust_10",
        HSz_DecTrust == 11 ~ "HSz_DecTrust_11", HSz_DecTrust ==
            12 ~ "HSz_DecTrust_12", HSz_DecTrust == 13 ~ "HSz_DecTrust_13",
        HSz_DecTrust == 14 ~ "HSz_DecTrust_14", HSz_DecTrust ==
```

```
            15 ~ "HSz_DecTrust_15", HSz_DecTrust == 16 ~ "HSz_DecTrust_16",
        HSz_DecTrust == 17 ~ "HSz_DecTrust_17", HSz_DecTrust ==
            18 ~ "HSz_DecTrust_18", HSz_DecTrust == 19 ~ "HSz_DecTrust_19",
        HSz_DecTrust == 20 ~ "HSz_DecTrust_20")) %>% pivot_wider(id_cols = ADz_PartcNum,
    names_from = HSz_DecTrust, values_from = DecTrust_Checked,
    values_fill = list(DecTrust_Checked = FALSE))
```

```
InfluenceWho <- Q1804dta %>% select(ADz_PartcNum, OPz_InfluenceWho) %>%
    separate_rows(OPz_InfluenceWho) %>% mutate(InfluenceWho_Checked = TRUE) %>%
    mutate(OPz_InfluenceWho = case_when(OPz_InfluenceWho == 1 ~
        "OPz_InfluenceWho_01", OPz_InfluenceWho == 2 ~ "OPz_InfluenceWho_02",
        OPz_InfluenceWho == 3 ~ "OPz_InfluenceWho_03", OPz_InfluenceWho ==
            4 ~ "OPz_InfluenceWho_04", OPz_InfluenceWho == 5 ~
            "OPz_InfluenceWho_05", OPz_InfluenceWho == 6 ~ "OPz_InfluenceWho_06",
        OPz_InfluenceWho == 7 ~ "OPz_InfluenceWho_07", OPz_InfluenceWho ==
            8 ~ "OPz_InfluenceWho_08", OPz_InfluenceWho == 9 ~
            "OPz_InfluenceWho_09", OPz_InfluenceWho == 10 ~ "OPz_InfluenceWho_10",
        OPz_InfluenceWho == 11 ~ "OPz_InfluenceWho_11", OPz_InfluenceWho ==
            12 ~ "OPz_InfluenceWho_12", OPz_InfluenceWho == 13 ~
            "OPz_InfluenceWho_13", OPz_InfluenceWho == 14 ~ "OPz_InfluenceWho_14",
        OPz_InfluenceWho == 15 ~ "OPz_InfluenceWho_15", OPz_InfluenceWho ==
            16 ~ "OPz_InfluenceWho_16", OPz_InfluenceWho == 17 ~
            "OPz_InfluenceWho_17", OPz_InfluenceWho == 18 ~ "OPz_InfluenceWho_18",
        OPz_InfluenceWho == 19 ~ "OPz_InfluenceWho_19", OPz_InfluenceWho ==
            20 ~ "OPz_InfluenceWho_20")) %>% pivot_wider(id_cols = ADz_PartcNum,
    names_from = OPz_InfluenceWho, values_from = InfluenceWho_Checked,
    values_fill = list(InfluenceWho_Checked = FALSE)) %>% select(-c("NA"))
```

```
NoScrnRsn <- Q1804dta %>% select(ADz_PartcNum, EXz_NoScrnRsn) %>%
    separate_rows(EXz_NoScrnRsn) %>% mutate(NoScrnRsn_Checked = TRUE) %>%
    mutate(EXz_NoScrnRsn = case_when(EXz_NoScrnRsn == 1 ~ "EXz_NoScrnRsn_01",
        EXz_NoScrnRsn == 2 ~ "EXz_NoScrnRsn_02", EXz_NoScrnRsn ==
            3 ~ "EXz_NoScrnRsn_03", EXz_NoScrnRsn == 4 ~ "EXz_NoScrnRsn_04",
        EXz_NoScrnRsn == 5 ~ "EXz_NoScrnRsn_05", EXz_NoScrnRsn ==
            6 ~ "EXz_NoScrnRsn_06", EXz_NoScrnRsn == 7 ~ "EXz_NoScrnRsn_07",
        EXz_NoScrnRsn == 8 ~ "EXz_NoScrnRsn_08", EXz_NoScrnRsn ==
            9 ~ "EXz_NoScrnRsn_09", EXz_NoScrnRsn == 10 ~ "EXz_NoScrnRsn_10",
        EXz_NoScrnRsn == 11 ~ "EXz_NoScrnRsn_11", EXz_NoScrnRsn ==
```

```
            12 ~ "EXz_NoScrnRsn_12", EXz_NoScrnRsn == 13 ~ "EXz_NoScrnRsn_13",
        EXz_NoScrnRsn == 14 ~ "EXz_NoScrnRsn_14", EXz_NoScrnRsn ==
            15 ~ "EXz_NoScrnRsn_15", EXz_NoScrnRsn == 16 ~ "EXz_NoScrnRsn_16",
        EXz_NoScrnRsn == 17 ~ "EXz_NoScrnRsn_17", EXz_NoScrnRsn ==
            18 ~ "EXz_NoScrnRsn_18", EXz_NoScrnRsn == 19 ~ "EXz_NoScrnRsn_19",
        EXz_NoScrnRsn == 20 ~ "EXz_NoScrnRsn_20", EXz_NoScrnRsn ==
            NA ~ "NA")) %>% pivot_wider(id_cols = ADz_PartcNum,
    names_from = EXz_NoScrnRsn, values_from = NoScrnRsn_Checked,
    values_fill = list(NoScrnRsn_Checked = FALSE)) %>% select(-c("NA"))
```

```
TreatNOWhy <- Q1804dta %>% select(ADz_PartcNum, EXz_TreatNOWhy) %>%
    separate_rows(EXz_TreatNOWhy) %>% mutate(TreatNOWhy_Checked = TRUE) %>%
    mutate(EXz_TreatNOWhy = case_when(EXz_TreatNOWhy == 1 ~ "EXz_TreatNOWhy_01",
        EXz_TreatNOWhy == 2 ~ "EXz_TreatNOWhy_02", EXz_TreatNOWhy ==
            3 ~ "EXz_TreatNOWhy_03", EXz_TreatNOWhy == 4 ~ "EXz_TreatNOWhy_04",
        EXz_TreatNOWhy == 5 ~ "EXz_TreatNOWhy_05", EXz_TreatNOWhy ==
            6 ~ "EXz_TreatNOWhy_06", EXz_TreatNOWhy == 7 ~ "EXz_TreatNOWhy_07",
        EXz_TreatNOWhy == 8 ~ "EXz_TreatNOWhy_08", EXz_TreatNOWhy ==
            9 ~ "EXz_TreatNOWhy_09", EXz_TreatNOWhy == 10 ~ "EXz_TreatNOWhy_10",
        EXz_TreatNOWhy == 11 ~ "EXz_TreatNOWhy_11", EXz_TreatNOWhy ==
            12 ~ "EXz_TreatNOWhy_12", EXz_TreatNOWhy == 13 ~
            "EXz_TreatNOWhy_13", EXz_TreatNOWhy == 14 ~ "EXz_TreatNOWhy_14",
        EXz_TreatNOWhy == 15 ~ "EXz_TreatNOWhy_15", EXz_TreatNOWhy ==
            16 ~ "EXz_TreatNOWhy_16", EXz_TreatNOWhy == 17 ~
            "EXz_TreatNOWhy_17", EXz_TreatNOWhy == 18 ~ "EXz_TreatNOWhy_18",
        EXz_TreatNOWhy == 19 ~ "EXz_TreatNOWhy_19", EXz_TreatNOWhy ==
            20 ~ "EXz_TreatNOWhy_20")) %>% pivot_wider(id_cols = ADz_PartcNum,
    names_from = EXz_TreatNOWhy, values_from = TreatNOWhy_Checked,
    values_fill = list(TreatNOWhy_Checked = FALSE)) %>% select(-c("NA"))
```

##Combine all expanded multi-select questions into the master dataset

```
Q1804dta_MSJoin <- Q1804dta
Q1804dta_MSJoin <- left_join(Q1804dta_MSJoin, LangPref, by = "ADz_PartcNum")
Q1804dta_MSJoin <- left_join(Q1804dta_MSJoin, ServLang, by = "ADz_PartcNum")
Q1804dta_MSJoin <- left_join(Q1804dta_MSJoin, LangInterp, by = "ADz_PartcNum")
Q1804dta_MSJoin <- left_join(Q1804dta_MSJoin, CarePay, by = "ADz_PartcNum")
Q1804dta_MSJoin <- left_join(Q1804dta_MSJoin, ProvTyp, by = "ADz_PartcNum")
```

```
Q1804dta_MSJoin <- left_join(Q1804dta_MSJoin, Bsis, by = "ADz_PartcNum")
Q1804dta_MSJoin <- left_join(Q1804dta_MSJoin, ScrnWhy, by = "ADz_PartcNum")
Q1804dta_MSJoin <- left_join(Q1804dta_MSJoin, WHCDecis, by = "ADz_PartcNum")
Q1804dta_MSJoin <- left_join(Q1804dta_MSJoin, DecTrust, by = "ADz_PartcNum")
Q1804dta_MSJoin <- left_join(Q1804dta_MSJoin, InfluenceWho, by = "ADz_PartcNum")
Q1804dta_MSJoin <- left_join(Q1804dta_MSJoin, NoScrnRsn, by = "ADz_PartcNum")
Q1804dta_MSJoin <- left_join(Q1804dta_MSJoin, TreatNOWhy, by = "ADz_PartcNum")
```

##Rename the factors of the multi-select variables

```
Q1804dta_MSJoin <- Q1804dta_MSJoin %>% mutate(SDz_LangPrefAll_01 = case_when(SDz_LangPrefAll_01 ==
    TRUE ~ "Yes", SDz_LangPrefAll_01 == FALSE ~ "No")) %>% mutate(SDz_LangPrefAll_02 = case_when(SDz_LangPrefAll_02 ==
    TRUE ~ "Yes", SDz_LangPrefAll_02 == FALSE ~ "No")) %>% mutate(SDz_LangPrefAll_06 = case_when(SDz_LangPrefAll_06 ==
    TRUE ~ "Yes", SDz_LangPrefAll_06 == FALSE ~ "No")) %>% mutate(SDz_LangPrefAll_07 = case_when(SDz_LangPrefAll_07 ==
    TRUE ~ "Yes", SDz_LangPrefAll_07 == FALSE ~ "No")) %>% mutate(HSz_ServLangAll_01 = case_when(HSz_ServLangAll_01 ==
    TRUE ~ "Yes", HSz_ServLangAll_01 == FALSE ~ "No")) %>% mutate(HSz_ServLangAll_02 = case_when(HSz_ServLangAll_02 ==
    TRUE ~ "Yes", HSz_ServLangAll_02 == FALSE ~ "No")) %>% mutate(HSz_ServLangAll_06 = case_when(HSz_ServLangAll_06 ==
    TRUE ~ "Yes", HSz_ServLangAll_06 == FALSE ~ "No")) %>% mutate(HSz_ServLangAll_07 = case_when(HSz_ServLangAll_07 ==
    TRUE ~ "Yes", HSz_ServLangAll_07 == FALSE ~ "No")) %>% mutate(HSz_LangInterpAll_06 = case_when(HSz_LangInterpAll_06 ==
    TRUE ~ "Yes", HSz_LangInterpAll_06 == FALSE ~ "No")) %>%
    mutate(HSz_LangInterpAll_07 = case_when(HSz_LangInterpAll_07 ==
        TRUE ~ "No", HSz_LangInterpAll_07 == FALSE ~ "Yes")) %>%
    mutate(HSz_LangInterpAll_02 = case_when(HSz_LangInterpAll_02 ==
        TRUE ~ "Yes", HSz_LangInterpAll_02 == FALSE ~ "No")) %>%
    mutate(BAF_CarePayAll_01 = case_when(BAF_CarePayAll_01 ==
        TRUE ~ "Yes", BAF_CarePayAll_01 == FALSE ~ "No")) %>%
    mutate(BAF_CarePayAll_08 = case_when(BAF_CarePayAll_08 ==
        TRUE ~ "Yes", BAF_CarePayAll_08 == FALSE ~ "No")) %>%
    mutate(BAF_CarePayAll_02 = case_when(BAF_CarePayAll_02 ==
        TRUE ~ "Yes", BAF_CarePayAll_02 == FALSE ~ "No")) %>%
    mutate(BAF_CarePayAll_04 = case_when(BAF_CarePayAll_04 ==
        TRUE ~ "Yes", BAF_CarePayAll_04 == FALSE ~ "No")) %>%
    mutate(BAF_CarePayAll_10 = case_when(BAF_CarePayAll_10 ==
        TRUE ~ "Yes", BAF_CarePayAll_10 == FALSE ~ "No")) %>%
    mutate(BAF_CarePayAll_09 = case_when(BAF_CarePayAll_09 ==
        TRUE ~ "Yes", BAF_CarePayAll_09 == FALSE ~ "No")) %>%
    mutate(GNz_WHCDecisAll_01 = case_when(GNz_WHCDecisAll_01 ==
        TRUE ~ "Yes", GNz_WHCDecisAll_01 == FALSE ~ "No")) %>%
```

```
    mutate(GNz_WHCDecisAll_02 = case_when(GNz_WHCDecisAll_02 ==
        TRUE ~ "Yes", GNz_WHCDecisAll_02 == FALSE ~ "No")) %>%
    mutate(HSz_DecTrust_02 = case_when(HSz_DecTrust_02 == TRUE ~
        "Yes", HSz_DecTrust_02 == FALSE ~ "No")) %>% mutate(HSz_DecTrust_07 = case_when(HSz_DecTrust_07 ==
    TRUE ~ "Yes", HSz_DecTrust_07 == FALSE ~ "No")) %>% mutate(HSz_DecTrust_04 = case_when(HSz_DecTrust_04 ==
    TRUE ~ "Yes", HSz_DecTrust_04 == FALSE ~ "No")) %>% mutate(HSz_DecTrust_06 = case_when(HSz_DecTrust_06 ==
    TRUE ~ "Yes", HSz_DecTrust_06 == FALSE ~ "No")) %>% mutate(HSz_DecTrust_08 = case_when(HSz_DecTrust_08 ==
    TRUE ~ "Yes", HSz_DecTrust_08 == FALSE ~ "No")) %>% mutate(HSz_DecTrust_03 = case_when(HSz_DecTrust_03 ==
    TRUE ~ "Yes", HSz_DecTrust_03 == FALSE ~ "No")) %>% mutate(HSz_DecTrust_01 = case_when(HSz_DecTrust_01 ==
    TRUE ~ "Yes", HSz_DecTrust_01 == FALSE ~ "No")) %>% mutate(OPz_InfluenceWho_04 = case_when(OPz_InfluenceWho_04 ==
    TRUE ~ "Yes", OPz_InfluenceWho_04 == FALSE ~ "No")) %>% mutate(OPz_InfluenceWho_01 = case_when(OPz_InfluenceWho_01 ==
    TRUE ~ "Yes", OPz_InfluenceWho_01 == FALSE ~ "No")) %>% mutate(OPz_InfluenceWho_02 = case_when(OPz_InfluenceWho_02 ==
    TRUE ~ "Yes", OPz_InfluenceWho_02 == FALSE ~ "No")) %>% mutate(OPz_InfluenceWho_09 = case_when(OPz_InfluenceWho_09 ==
    TRUE ~ "Yes", OPz_InfluenceWho_09 == FALSE ~ "No")) %>% mutate(OPz_InfluenceWho_06 = case_when(OPz_InfluenceWho_06 ==
    TRUE ~ "Yes", OPz_InfluenceWho_06 == FALSE ~ "No"))
```

#Table 1. Demographics by gender

```
Demographics <- Q1804dta_MSJoin %>% select(SDz_Gender, SDz_Age,
    SDz_Educatn, SCz_MaritalStat, SDz_LangPrefAll_01, SDz_LangPrefAll_02,
    SDz_LangPrefAll_06, SDz_LangPrefAll_07, BEz_Screend) %>%
    mutate(SDz_Gender = case_when(SDz_Gender == 0 ~ "Female",
        SDz_Gender == 1 ~ "Male")) %>% mutate(SDz_Educatn = case_when(SDz_Educatn ==
    0 ~ "0) None", SDz_Educatn == 1 ~ "1) Quranic School", SDz_Educatn ==
    2 ~ "2) Primary education", SDz_Educatn == 3 ~ "3) Secondary school through university",
    SDz_Educatn == 4 ~ "3) Secondary school through university",
    SDz_Educatn == 5 ~ "3) Secondary school through university",
    SDz_Educatn == 99 ~ "Do Not Know / Not Sure")) %>% mutate(SCz_MaritalStat = case_when(SCz_MaritalStat ==
    1 ~ "1) Single, divorced, separated, or widowed", SCz_MaritalStat ==
    2 ~ "2) Married (monogamous household)", SCz_MaritalStat ==
    3 ~ "3) Married (polygamous household)", SCz_MaritalStat ==
    4 ~ "4) Living as married / cohabitating", SCz_MaritalStat ==
    5 ~ "1) Single, divorced, separated, or widowed", SCz_MaritalStat ==
    6 ~ "1) Single, divorced, separated, or widowed", SCz_MaritalStat ==
    99 ~ "Do Not Know / Not Sure")) %>% mutate(BEz_Screend = case_when(BEz_Screend ==
    0 ~ "0) Never screened", BEz_Screend == 1 ~ "1) One time only",
    BEz_Screend == 2 ~ "2) More than one time", BEz_Screend ==
        99 ~ "Do Not Know / Not Sure"))
```

```r
TA_DemographicsGen <- tableby(SDz_Gender ~ anova(SDz_Age, "meansd",
    "range") + fe(SDz_Educatn, "countpct") + fe(SCz_MaritalStat,
    "countpct") + fe(SDz_LangPrefAll_01, "countpct") + fe(SDz_LangPrefAll_02,
    "countpct") + fe(SDz_LangPrefAll_06, "countpct") + fe(SDz_LangPrefAll_07,
    "countpct") + fe(BEz_Screend, "countpct"), simulate.p.value = TRUE,
    data = Demographics)

labels(TA_DemographicsGen) <- c(SDz_Gender = "Gender", SDz_Age = "Age in years",
    SDz_Educatn = "Education level", SCz_MaritalStat = "Marital status",
    BEz_Screend = "Screened for cervical cancer", SDz_LangPrefAll_01 = "Malinke speaker",
    SDz_LangPrefAll_02 = "Pulaar speaker", SDz_LangPrefAll_06 = "Wolof speaker",
    SDz_LangPrefAll_07 = "French speaker")

summary(TA_DemographicsGen, title = "Arsenal Table 18Q04", pfootnote = TRUE)
```

```
##
## Table: Arsenal Table 18Q04
##
## |                                                              | Female (N=101)  |   Male (N=57)   |  Total (N=158)  |    p value|
## |:-------------------------------------------------------------|:---------------:|:---------------:|:---------------:|----------:|
## |**Age in years**                                              |                 |                 |                 |   0.006^1^|
## |   Mean (SD)                                   | 40.168 (8.631)  | 44.140 (8.355)  | 41.601 (8.718)  |           |
## |   Range                                       | 30.000 - 59.000 | 30.000 - 59.000 | 30.000 - 59.000 |           |
## |**Education level**                                           |                 |                 |                 | < 0.001^2^|
## |   0) None                                     |   26 (25.7%)    |    5 (8.9%)     |   31 (19.7%)    |           |
## |   1) Quranic School                           |   35 (34.7%)    |   21 (37.5%)    |   56 (35.7%)    |           |
## |   2) Primary education                        |   37 (36.6%)    |   17 (30.4%)    |   54 (34.4%)    |           |
## |   3) Secondary school through university      |    3 (3.0%)     |   13 (23.2%)    |   16 (10.2%)    |           |
## |**Marital status**                                            |                 |                 |                 |   0.068^2^|
## |   1) Single, divorced, separated, or widowed |    8 (7.9%)     |    3 (5.3%)     |    11 (7.0%)    |           |
## |   2) Married (monogamous household)           |   41 (40.6%)    |   34 (59.6%)    |   75 (47.5%)    |           |
## |   3) Married (polygamous household)           |   52 (51.5%)    |   20 (35.1%)    |   72 (45.6%)    |           |
## |**Malinke speaker**                                           |                 |                 |                 |   0.733^3^|
## |   No                                          |   39 (38.6%)    |   20 (35.1%)    |   59 (37.3%)    |           |
## |   Yes                                         |   62 (61.4%)    |   37 (64.9%)    |   99 (62.7%)    |           |
## |**Pulaar speaker**                                            |                 |                 |                 |   0.019^3^|
## |   No                                          |   48 (47.5%)    |   16 (28.1%)    |   64 (40.5%)    |           |
## |   Yes                                         |   53 (52.5%)    |   41 (71.9%)    |   94 (59.5%)    |           |
```

```
## |**Wolof speaker**                               |                |                |                |   0.039^3^|
## |   No                            |   80 (79.2%)   |   36 (63.2%)   |   116 (73.4%)  |           |
## |   Yes                           |   21 (20.8%)   |   21 (36.8%)   |   42 (26.6%)   |           |
## |**French speaker**                              |                |                |                |   0.004^3^|
## |   No                            |   78 (77.2%)   |   31 (54.4%)   |   109 (69.0%)  |           |
## |   Yes                           |   23 (22.8%)   |   26 (45.6%)   |   49 (31.0%)   |           |
## |**Screened for cervical cancer**                |                |                |                |           |
## |   0) Never screened             |   85 (84.2%)   |       0        |   85 (84.2%)   |           |
## |   1) One time only              |   14 (13.9%)   |       0        |   14 (13.9%)   |           |
## |   2) More than one time         |    2 (2.0%)    |       0        |    2 (2.0%)    |           |
## 1. Linear Model ANOVA
## 2. Fisher's Exact Test for Count Data with simulated p-value
##    (based on 2000 replicates)
## 3. Fisher's Exact Test for Count Data
```

```
# write2pdf(TA_DemographicsGen,
# here('Tables','T01_Demographics.pdf'))
# write2word(TA_DemographicsGen,
# here('Tables','T01_Demographics.doc'))
```

#Table 2. Perception of gender roles by gender

```
Power_Gendr <- Q1804dta_MSJoin %>% select(SDz_Gender, OPz_ScrnWRec,
    OPz_ScrnRecOthrs, GNz_WRole, GNz_ManDecis, OPz_InfluenceWho_01,
    OPz_InfluenceWho_04, OPz_InfluenceWho_02, OPz_InfluenceWho_06,
    OPz_InfluenceWho_09, HSz_DecTrust_02, HSz_DecTrust_07, HSz_DecTrust_04,
    HSz_DecTrust_06, HSz_DecTrust_08, HSz_DecTrust_03, HSz_DecTrust_01,
    GNz_WHCDecisAll_01, GNz_WHCDecisAll_02) %>% mutate(SDz_Gender = case_when(SDz_Gender ==
    0 ~ "0) Female", SDz_Gender == 1 ~ "1) Male")) %>% mutate(OPz_ScrnWRec = case_when(OPz_ScrnWRec ==
    1 ~ "1) Strongly Disagree", OPz_ScrnWRec == 2 ~ "2) Disagree",
    OPz_ScrnWRec == 3 ~ "3) Undecided", OPz_ScrnWRec == 4 ~ "4) Agree",
    OPz_ScrnWRec == 5 ~ "5) Strongly Agree")) %>% mutate(OPz_ScrnRecOthrs = case_when(OPz_ScrnRecOthrs ==
    1 ~ "1) Strongly Disagree", OPz_ScrnRecOthrs == 2 ~ "2) Disagree",
    OPz_ScrnRecOthrs == 3 ~ "3) Undecided", OPz_ScrnRecOthrs ==
        4 ~ "4) Agree", OPz_ScrnRecOthrs == 5 ~ "5) Strongly Agree")) %>%
    mutate(GNz_WRole = case_when(GNz_WRole == 1 ~ "1) Strongly Disagree",
        GNz_WRole == 2 ~ "2) Disagree", GNz_WRole == 3 ~ "3) Undecided",
        GNz_WRole == 4 ~ "4) Agree", GNz_WRole == 5 ~ "5) Strongly Agree")) %>%
```

```
    mutate(GNz_ManDecis = case_when(GNz_ManDecis == 1 ~ "1) Strongly Disagree",
        GNz_ManDecis == 2 ~ "2) Disagree", GNz_ManDecis == 3 ~
            "3) Undecided", GNz_ManDecis == 4 ~ "4) Agree", GNz_ManDecis ==
            5 ~ "5) Strongly Agree"))

TA_Power_Gendr <- tableby(SDz_Gender ~ fe(GNz_WRole, "countpct") +
    fe(GNz_ManDecis, "countpct"), simulate.p.value = TRUE, data = Power_Gendr)

labels(TA_Power_Gendr) <- c(GNz_WRole = "A womans most important role is to take care of her home and cook for her family",
    GNz_ManDecis = "A man should have the final word about decisions in his home")

summary(TA_Power_Gendr, title = "Arsenal Table 18Q04", pfootnote = TRUE)
```

```
##
## Table: Arsenal Table 18Q04
##
## |                                                                           | 0) Female (N=101) | 1) Male (N=57) | Total (N=1
## |:--------------------------------------------------------------------------|:-----------------:|:--------------:|:----------
## |**A womans most important role is to take care of her home and cook for her family** |                   |                |
## |   1) Strongly Disagree                                     |     6 (6.0%)      |    1 (1.8%)    |   7 (4.5%)
## |   2) Disagree                                              |    26 (26.0%)     |   6 (10.5%)    |  32 (20.4%
## |   3) Undecided                                             |     2 (2.0%)      |    2 (3.5%)    |   4 (2.5%)
## |   4) Agree                                                 |     7 (7.0%)      |   25 (43.9%)   |  32 (20.4%
## |   5) Strongly Agree                                        |    59 (59.0%)     |   23 (40.4%)   |  82 (52.2%
## |**A man should have the final word about decisions in his home**           |                   |                |
## |   1) Strongly Disagree                                     |     7 (6.9%)      |    1 (1.8%)    |   8 (5.1%)
## |   2) Disagree                                              |    24 (23.8%)     |    3 (5.4%)    |  27 (17.2%
## |   3) Undecided                                             |     1 (1.0%)      |    0 (0.0%)    |   1 (0.6%)
## |   4) Agree                                                 |    15 (14.9%)     |   13 (23.2%)   |  28 (17.8%
## |   5) Strongly Agree                                        |    54 (53.5%)     |   39 (69.6%)   |  93 (59.2%
## 1. Fisher's Exact Test for Count Data with simulated p-value
##    (based on 2000 replicates)
```

```
# write2pdf(TA_Power_Gendr,
# here('Tables','T02_Power_Gendr.pdf'))
# write2word(TA_Power_Gendr,
# here('Tables','T02_Power_Gendr.doc'))
# write2html(TA_Power_Gendr,
```

```
# here('Tables','T02_Power_Gendr.html'))
```

#Table 3. Adapted everyday discrimination scale by gender

```
Discr_Gendr <- Q1804dta_MSJoin %>% select(SDz_Gender, DSz_RespGen,
    DSz_RespPrtn, DSz_Smrt, DSz_Hnst, DSz_Thret) %>% mutate(SDz_Gender = case_when(SDz_Gender ==
    0 ~ "0) Female", SDz_Gender == 1 ~ "1) Male")) %>% mutate(DSz_RespGen = case_when(DSz_RespGen ==
    1 ~ "1) Every day", DSz_RespGen == 2 ~ "2) Every week", DSz_RespGen ==
    3 ~ "3) A few times per year", DSz_RespGen == 4 ~ "4) A few times in my life",
    DSz_RespGen == 5 ~ "5) Never", DSz_RespGen == 99 ~ "Do Not Know / Not Sure")) %>%
    mutate(DSz_RespPrtn = case_when(DSz_RespPrtn == 1 ~ "1) Every day",
        DSz_RespPrtn == 2 ~ "2) Every week", DSz_RespPrtn ==
            3 ~ "3) A few times per year", DSz_RespPrtn == 4 ~
            "4) A few times in my life", DSz_RespPrtn == 5 ~
            "5) Never", DSz_RespPrtn == 99 ~ "Do Not Know / Not Sure")) %>%
    mutate(DSz_Smrt = case_when(DSz_Smrt == 1 ~ "1) Every day",
        DSz_Smrt == 2 ~ "2) Every week", DSz_Smrt == 3 ~ "3) A few times per year",
        DSz_Smrt == 4 ~ "4) A few times in my life", DSz_Smrt ==
            5 ~ "5) Never", DSz_Smrt == 99 ~ "Do Not Know / Not Sure")) %>%
    mutate(DSz_Hnst = case_when(DSz_Hnst == 1 ~ "1) Every day",
        DSz_Hnst == 2 ~ "2) Every week", DSz_Hnst == 3 ~ "3) A few times per year",
        DSz_Hnst == 4 ~ "4) A few times in my life", DSz_Hnst ==
            5 ~ "5) Never", DSz_Hnst == 99 ~ "Do Not Know / Not Sure")) %>%
    mutate(DSz_Thret = case_when(DSz_Thret == 1 ~ "1) Every day",
        DSz_Thret == 2 ~ "2) Every week", DSz_Thret == 3 ~ "3) A few times per year",
        DSz_Thret == 4 ~ "4) A few times in my life", DSz_Thret ==
            5 ~ "5) Never", DSz_Thret == 99 ~ "Do Not Know / Not Sure"))

TA_Discr_Gendr <- tableby(SDz_Gender ~ fe(DSz_RespGen, "countpct") +
    fe(DSz_RespPrtn, "countpct") + fe(DSz_Smrt, "countpct") +
    fe(DSz_Hnst, "countpct") + fe(DSz_Thret, "countpct"), simulate.p.value = TRUE,
    data = Discr_Gendr)

labels(TA_Discr_Gendr) <- c(DSz_RespGen = "Feel treated with less courtesy or respect than others",
    DSz_RespPrtn = "Feel treated with less courtesy or respect by their spouse",
    DSz_Smrt = "Feel that others act as if they are not smart",
    DSz_Hnst = "Feel perceived as being dishonest", DSz_Thret = "Feel threatened by others")
```

```r
summary(TA_Discr_Gendr, title = "Arsenal Table 18Q04", pfootnote = TRUE)
```

```
##
## Table: Arsenal Table 18Q04
##
## |                                                                | 0) Female (N=101) | 1) Male (N=57) | Total (N=158) |   p value|
## |:---------------------------------------------------------------|:-----------------:|:--------------:|:-------------:|---------:|
## |**Feel treated with less courtesy or respect than others**      |                   |                |               | 0.084^1^|
## |   1) Every day                                  |     9 (9.2%)      |    0 (0.0%)    |   9 (5.9%)    |          |
## |   2) Every week                                 |     2 (2.0%)      |    0 (0.0%)    |   2 (1.3%)    |          |
## |   3) A few times per year                       |     6 (6.1%)      |    3 (5.6%)    |   9 (5.9%)    |          |
## |   4) A few times in my life                     |    22 (22.4%)     |   10 (18.5%)   |  32 (21.1%)   |          |
## |   5) Never                                      |    59 (60.2%)     |   41 (75.9%)   |  100 (65.8%)  |          |
## |**Feel treated with less courtesy or respect by their spouse** |                   |                |               | < 0.001^1^|
## |   1) Every day                                  |    14 (14.3%)     |    0 (0.0%)    |   14 (9.3%)   |          |
## |   2) Every week                                 |     4 (4.1%)      |    0 (0.0%)    |   4 (2.7%)    |          |
## |   3) A few times per year                       |    15 (15.3%)     |    2 (3.8%)    |  17 (11.3%)   |          |
## |   4) A few times in my life                     |    18 (18.4%)     |   9 (17.3%)    |  27 (18.0%)   |          |
## |   5) Never                                      |    47 (48.0%)     |   41 (78.8%)   |  88 (58.7%)   |          |
## |**Feel that others act as if they are not smart**              |                   |                |               | 0.032^1^|
## |   1) Every day                                  |     1 (1.0%)      |    0 (0.0%)    |   1 (0.6%)    |          |
## |   2) Every week                                 |     1 (1.0%)      |    0 (0.0%)    |   1 (0.6%)    |          |
## |   3) A few times per year                       |     4 (4.0%)      |    5 (9.4%)    |   9 (5.8%)    |          |
## |   4) A few times in my life                     |    16 (15.8%)     |   17 (32.1%)   |  33 (21.4%)   |          |
## |   5) Never                                      |    79 (78.2%)     |   31 (58.5%)   |  110 (71.4%)  |          |
## |**Feel perceived as being dishonest**                           |                   |                |               | 0.105^1^|
## |   3) A few times per year                       |     1 (1.0%)      |    2 (4.2%)    |   3 (2.0%)    |          |
## |   4) A few times in my life                     |    11 (11.0%)     |   10 (20.8%)   |  21 (14.2%)   |          |
## |   5) Never                                      |    88 (88.0%)     |   36 (75.0%)   |  124 (83.8%)  |          |
## |**Feel threatened by others**                                   |                   |                |               | 0.436^1^|
## |   1) Every day                                  |     1 (1.0%)      |    0 (0.0%)    |   1 (0.7%)    |          |
## |   3) A few times per year                       |     3 (3.0%)      |    3 (6.1%)    |   6 (4.1%)    |          |
## |   4) A few times in my life                     |    16 (16.2%)     |    4 (8.2%)    |  20 (13.5%)   |          |
## |   5) Never                                      |    79 (79.8%)     |   42 (85.7%)   |  121 (81.8%)  |          |
## 1. Fisher's Exact Test for Count Data with simulated p-value
##    (based on 2000 replicates)
```

```
# write2pdf(TA_Discr_Gendr,
# here('Tables','T03_Discr_Gendr.pdf'))
# write2word(TA_Discr_Gendr,
# here('Tables','T03_Discr_Gendr.doc'))
# write2html(TA_Discr_Gendr,
# here('Tables','T03_Discr_Gendr.html'))
```

#Table 4. Adapted cancer stigma scale by gender

```
Stgma_Gendr <- Q1804dta_MSJoin %>% select(SDz_Gender, STz_CAAbnl,
    STz_CAComfNear, STz_CANeedsPriority, STz_CAPtFault, STz_CAFeelPity,
    STz_CADxFear, OPz_CAWorry) %>% mutate(SDz_Gender = case_when(SDz_Gender ==
    0 ~ "0) Female", SDz_Gender == 1 ~ "1) Male")) %>% mutate(STz_CAAbnl = case_when(STz_CAAbnl ==
    1 ~ "1) Strongly Disagree", STz_CAAbnl == 2 ~ "2) Disagree",
    STz_CAAbnl == 3 ~ "3) Undecided", STz_CAAbnl == 4 ~ "4) Agree",
    STz_CAAbnl == 5 ~ "5) Strongly Agree")) %>% mutate(STz_CAComfNear = case_when(STz_CAComfNear ==
    1 ~ "1) Strongly Disagree", STz_CAComfNear == 2 ~ "2) Disagree",
    STz_CAComfNear == 3 ~ "3) Undecided", STz_CAComfNear == 4 ~
        "4) Agree", STz_CAComfNear == 5 ~ "5) Strongly Agree")) %>%
    mutate(STz_CANeedsPriority = case_when(STz_CANeedsPriority ==
        1 ~ "1) Strongly Disagree", STz_CANeedsPriority == 2 ~
        "2) Disagree", STz_CANeedsPriority == 3 ~ "3) Undecided",
        STz_CANeedsPriority == 4 ~ "4) Agree", STz_CANeedsPriority ==
            5 ~ "5) Strongly Agree")) %>% mutate(STz_CAPtFault = case_when(STz_CAPtFault ==
    1 ~ "1) Strongly Disagree", STz_CAPtFault == 2 ~ "2) Disagree",
    STz_CAPtFault == 3 ~ "3) Undecided", STz_CAPtFault == 4 ~
        "4) Agree", STz_CAPtFault == 5 ~ "5) Strongly Agree")) %>%
    mutate(STz_CAFeelPity = case_when(STz_CAFeelPity == 1 ~ "1) Strongly Disagree",
        STz_CAFeelPity == 2 ~ "2) Disagree", STz_CAFeelPity ==
            3 ~ "3) Undecided", STz_CAFeelPity == 4 ~ "4) Agree",
        STz_CAFeelPity == 5 ~ "5) Strongly Agree")) %>% mutate(STz_CADxFear = case_when(STz_CADxFear ==
    1 ~ "1) Strongly Disagree", STz_CADxFear == 2 ~ "2) Disagree",
    STz_CADxFear == 3 ~ "3) Undecided", STz_CADxFear == 4 ~ "4) Agree",
    STz_CADxFear == 5 ~ "5) Strongly Agree")) %>% mutate(OPz_CAWorry = case_when(OPz_CAWorry ==
    1 ~ "1) Strongly Disagree", OPz_CAWorry == 2 ~ "2) Disagree",
    OPz_CAWorry == 3 ~ "3) Undecided", OPz_CAWorry == 4 ~ "4) Agree",
    OPz_CAWorry == 5 ~ "5) Strongly Agree"))
```

```r
TA_Stgma_Gendr <- tableby(SDz_Gender ~ fe(STz_CAComfNear, "countpct") +
    fe(STz_CAAbnl, "countpct") + fe(STz_CANeedsPriority, "countpct") +
    fe(STz_CAPtFault, "countpct") + fe(STz_CAFeelPity, "countpct") +
    fe(STz_CADxFear, "countpct") + fe(OPz_CAWorry, "countpct"),
    simulate.p.value = TRUE, data = Stgma_Gendr)

labels(TA_Stgma_Gendr) <- c(STz_CAAbnl = "Once youve had cancer youre never normal again.",
    STz_CAComfNear = "I would not feel comfortable around someone with cancer.",
    STz_CANeedsPriority = "The health care needs of people with cancer should not be prioritized.",
    STz_CAPtFault = "If a person has cancer its probably their fault.",
    STz_CAFeelPity = " I would feel sorry for someone with cancer.",
    STz_CADxFear = "I feel that cancer is more frightening than most other diseases.",
    OPz_CAWorry = "Other women often state that they are worried about getting cancer.")

summary(TA_Stgma_Gendr, title = "Arsenal Table 18Q04", pfootnote = TRUE)
```

```
##
## Table: Arsenal Table 18Q04
##
## |                                                                          | 0) Female (N=101) | 1) Male (N=57) | Total (N=158) |    p
## |:-------------------------------------------------------------------------|:-----------------:|:--------------:|:-------------:|:-----
## |**I would not feel comfortable around someone with cancer.**              |                   |                |               | < 0.
## |   1) Strongly Disagree                                    |    11 (11.0%)     |   11 (19.6%)   |  22 (14.1%)   |
## |   2) Disagree                                             |    44 (44.0%)     |   31 (55.4%)   |  75 (48.1%)   |
## |   3) Undecided                                            |     1 (1.0%)      |    4 (7.1%)    |   5 (3.2%)    |
## |   4) Agree                                                |    19 (19.0%)     |   9 (16.1%)    |  28 (17.9%)   |
## |   5) Strongly Agree                                       |    25 (25.0%)     |    1 (1.8%)    |  26 (16.7%)   |
## |**Once youve had cancer youre never normal again.**                       |                   |                |               | < 0.
## |   1) Strongly Disagree                                    |     2 (2.0%)      |   7 (12.5%)    |   9 (5.8%)    |
## |   2) Disagree                                             |     8 (8.0%)      |   12 (21.4%)   |  20 (12.8%)   |
## |   3) Undecided                                            |     2 (2.0%)      |   9 (16.1%)    |   11 (7.1%)   |
## |   4) Agree                                                |    31 (31.0%)     |   23 (41.1%)   |  54 (34.6%)   |
## |   5) Strongly Agree                                       |    57 (57.0%)     |    5 (8.9%)    |  62 (39.7%)   |
## |**The health care needs of people with cancer should not be prioritized.** |                   |                |               | < 0.
## |   1) Strongly Disagree                                    |     9 (9.1%)      |   18 (32.1%)   |  27 (17.4%)   |
## |   2) Disagree                                             |    24 (24.2%)     |   31 (55.4%)   |  55 (35.5%)   |
## |   3) Undecided                                            |     3 (3.0%)      |    2 (3.6%)    |   5 (3.2%)    |
## |   4) Agree                                                |    31 (31.3%)     |    2 (3.6%)    |  33 (21.3%)   |
```

```
## |   5) Strongly Agree                                          |   32 (32.3%)     |    3 (5.4%)     |  35 (22.6%)    |
## |**If a person has cancer its probably their fault.**                     |                  |                 |                | < 0.
## |   1) Strongly Disagree                                   |   11 (11.0%)     |   11 (19.6%)    |  22 (14.1%)    |
## |   2) Disagree                                            |   40 (40.0%)     |   23 (41.1%)    |  63 (40.4%)    |
## |   3) Undecided                                           |    6 (6.0%)      |   14 (25.0%)    |  20 (12.8%)    |
## |   4) Agree                                               |   16 (16.0%)     |   7 (12.5%)     |  23 (14.7%)    |
## |   5) Strongly Agree                                      |   27 (27.0%)     |    1 (1.8%)     |  28 (17.9%)    |
## |**I would feel sorry for someone with cancer.**                          |                  |                 |                |   0.
## |   1) Strongly Disagree                                   |    1 (1.0%)      |    4 (7.1%)     |   5 (3.2%)     |
## |   2) Disagree                                            |    9 (9.0%)      |    3 (5.4%)     |   12 (7.7%)    |
## |   3) Undecided                                           |    0 (0.0%)      |    1 (1.8%)     |   1 (0.6%)     |
## |   4) Agree                                               |   48 (48.0%)     |   27 (48.2%)    |  75 (48.1%)    |
## |   5) Strongly Agree                                      |   42 (42.0%)     |   21 (37.5%)    |  63 (40.4%)    |
## |**I feel that cancer is more frightening than most other diseases.**     |                  |                 |                | < 0.
## |   1) Strongly Disagree                                   |    0 (0.0%)      |   6 (10.9%)     |   6 (3.9%)     |
## |   2) Disagree                                            |   11 (11.1%)     |   15 (27.3%)    |  26 (16.9%)    |
## |   3) Undecided                                           |    4 (4.0%)      |   5 (9.1%)      |   9 (5.8%)     |
## |   4) Agree                                               |   34 (34.3%)     |   20 (36.4%)    |  54 (35.1%)    |
## |   5) Strongly Agree                                      |   50 (50.5%)     |   9 (16.4%)     |  59 (38.3%)    |
## |**Other women often state that they are worried about getting cancer.**  |                  |                 |                | < 0.
## |   1) Strongly Disagree                                   |    0 (0.0%)      |    1 (1.8%)     |   1 (0.6%)     |
## |   2) Disagree                                            |    3 (3.0%)      |    1 (1.8%)     |   4 (2.6%)     |
## |   3) Undecided                                           |   24 (24.0%)     |   30 (53.6%)    |  54 (34.6%)    |
## |   4) Agree                                               |   25 (25.0%)     |   20 (35.7%)    |  45 (28.8%)    |
## |   5) Strongly Agree                                      |   48 (48.0%)     |    4 (7.1%)     |  52 (33.3%)    |
## 1. Fisher's Exact Test for Count Data with simulated p-value
##   (based on 2000 replicates)
```

```
# write2pdf(TA_Stgma_Gendr,
# here('Tables','T04_Stgma_Gendr.pdf'))
# write2word(TA_Stgma_Gendr,
# here('Tables','T04_Stgma_Gendr.doc'))
# write2html(TA_Stgma_Gendr,
# here('Tables','T04_Stgma_Gendr.html'))
```

#Table 5. Cancer Attitudes by gender

```
Attd_Gendr <- Q1804dta_MSJoin %>% select(SDz_Gender, BEz_Screend,
    ATz_BnfComf, ATz_MyDxSlfKno, ATz_MyDxFamKno, ATz_FamDxMyKno,
    ATz_DxFate) %>% mutate(SDz_Gender = case_when(SDz_Gender ==
    0 ~ "0) Female", SDz_Gender == 1 ~ "1) Male")) %>% mutate(ATz_BnfComf = case_when(ATz_BnfComf ==
    1 ~ "1) Strongly Disagree", ATz_BnfComf == 2 ~ "2) Disagree",
    ATz_BnfComf == 3 ~ "3) Undecided", ATz_BnfComf == 4 ~ "4) Agree",
    ATz_BnfComf == 5 ~ "5) Strongly Agree")) %>% mutate(ATz_MyDxSlfKno = case_when(ATz_MyDxSlfKno ==
    1 ~ "1) Strongly Disagree", ATz_MyDxSlfKno == 2 ~ "2) Disagree",
    ATz_MyDxSlfKno == 3 ~ "3) Undecided", ATz_MyDxSlfKno == 4 ~
        "4) Agree", ATz_MyDxSlfKno == 5 ~ "5) Strongly Agree")) %>%
    mutate(ATz_MyDxFamKno = case_when(ATz_MyDxFamKno == 1 ~ "1) Strongly Disagree",
        ATz_MyDxFamKno == 2 ~ "2) Disagree", ATz_MyDxFamKno ==
            3 ~ "3) Undecided", ATz_MyDxFamKno == 4 ~ "4) Agree",
        ATz_MyDxFamKno == 5 ~ "5) Strongly Agree")) %>% mutate(ATz_FamDxMyKno = case_when(ATz_FamDxMyKno ==
    1 ~ "1) Strongly Disagree", ATz_FamDxMyKno == 2 ~ "2) Disagree",
    ATz_FamDxMyKno == 3 ~ "3) Undecided", ATz_FamDxMyKno == 4 ~
        "4) Agree", ATz_FamDxMyKno == 5 ~ "5) Strongly Agree")) %>%
    mutate(ATz_DxFate = case_when(ATz_DxFate == 1 ~ "1) Strongly Disagree",
        ATz_DxFate == 2 ~ "2) Disagree", ATz_DxFate == 3 ~ "3) Undecided",
        ATz_DxFate == 4 ~ "4) Agree", ATz_DxFate == 5 ~ "5) Strongly Agree"))

TA_Attd_Gendr <- tableby(SDz_Gender ~ fe(ATz_BnfComf, "countpct") +
    fe(ATz_MyDxSlfKno, "countpct") + fe(ATz_MyDxFamKno, "countpct") +
    fe(ATz_FamDxMyKno, "countpct") + fe(ATz_DxFate, "countpct"),
    simulate.p.value = TRUE, data = Attd_Gendr)

labels(TA_Attd_Gendr) <- c(ATz_BnfComf = "Cancer testing or treatment that is unpleasant is worth getting if it would help me to live long
    ATz_MyDxSlfKno = "If I had cancer, I would want to know that I have it",
    ATz_MyDxFamKno = "If I had cancer, I would want my family to know that I have it.",
    ATz_FamDxMyKno = "If someone else in my family had cancer, I would want to know that they have it.",
    ATz_DxFate = "Getting a serious disease like cancer is fate, there is nothing I can do to change fate")

summary(TA_Attd_Gendr, title = "Arsenal Table 18Q04", pfootnote = TRUE)
```

```
##
## Table: Arsenal Table 18Q04
##
## |                                                                                                                   | 0) Female (N=101) | 1) Male (
```

```
## |:-------------------------------------------------------------------------------------------|:-----------------:|:---------
## |**Cancer testing or treatment that is unpleasant is worth getting if it would help me to live longer** |                   |
## |   1) Strongly Disagree                                                      |     2 (2.0%)      |    1 (1.9
## |   2) Disagree                                                               |     8 (8.0%)      |    0 (0.0
## |   3) Undecided                                                              |     9 (9.0%)      |   6 (11.1
## |   4) Agree                                                                  |    44 (44.0%)     |   34 (63.
## |   5) Strongly Agree                                                         |    37 (37.0%)     |   13 (24.
## |**If I had cancer, I would want to know that I have it**                                    |                   |
## |   1) Strongly Disagree                                                      |     1 (1.0%)      |    1 (1.8
## |   2) Disagree                                                               |     5 (5.1%)      |    0 (0.0
## |   3) Undecided                                                              |     0 (0.0%)      |    1 (1.8
## |   4) Agree                                                                  |    40 (40.4%)     |   17 (30.
## |   5) Strongly Agree                                                         |    53 (53.5%)     |   36 (65.
## |**If I had cancer, I would want my family to know that I have it.**                         |                   |
## |   1) Strongly Disagree                                                      |    10 (10.0%)     |    1 (1.8
## |   2) Disagree                                                               |    18 (18.0%)     |    2 (3.6
## |   3) Undecided                                                              |     3 (3.0%)      |    3 (5.4
## |   4) Agree                                                                  |    39 (39.0%)     |   17 (30.
## |   5) Strongly Agree                                                         |    30 (30.0%)     |   33 (58.
## |**If someone else in my family had cancer, I would want to know that they have it.**        |                   |
## |   1) Strongly Disagree                                                      |     4 (4.0%)      |    1 (1.8
## |   2) Disagree                                                               |    25 (25.3%)     |    0 (0.0
## |   4) Agree                                                                  |    38 (38.4%)     |   23 (41.
## |   5) Strongly Agree                                                         |    32 (32.3%)     |   32 (57.
## |**Getting a serious disease like cancer is fate, there is nothing I can do to change fate** |                   |
## |   1) Strongly Disagree                                                      |    24 (24.0%)     |   11 (19.
## |   2) Disagree                                                               |    27 (27.0%)     |   10 (17.
## |   3) Undecided                                                              |     6 (6.0%)      |   10 (17.
## |   4) Agree                                                                  |    30 (30.0%)     |   20 (35.
## |   5) Strongly Agree                                                         |    13 (13.0%)     |    5 (8.9
## 1. Fisher's Exact Test for Count Data with simulated p-value
##    (based on 2000 replicates)
```

```
# write2pdf(TA_Attd_Gendr,
# here('Tables','T05_Attd_Gendr.pdf'))
# write2word(TA_Attd_Gendr,
# here('Tables','T05_Attd_Gendr.doc'))
# write2html(TA_Attd_Gendr,
# here('Tables','T05_Attd_Gendr.html'))
```

#Table 6. Cervical cancer screening recommendation by gender

```
ScrnRec_Gendr <- Q1804dta_MSJoin %>% select(SDz_Gender, OPz_ScrnWRec,
    OPz_ScrnRecOthrs, GNz_WRole, GNz_ManDecis, OPz_InfluenceWho_01,
    OPz_InfluenceWho_04, OPz_InfluenceWho_02, OPz_InfluenceWho_06,
    OPz_InfluenceWho_09, HSz_DecTrust_02, HSz_DecTrust_07, HSz_DecTrust_04,
    HSz_DecTrust_06, HSz_DecTrust_08, HSz_DecTrust_03, HSz_DecTrust_01,
    GNz_WHCDecisAll_01, GNz_WHCDecisAll_02) %>% mutate(SDz_Gender = case_when(SDz_Gender ==
    0 ~ "0) Female", SDz_Gender == 1 ~ "1) Male")) %>% mutate(OPz_ScrnWRec = case_when(OPz_ScrnWRec ==
    1 ~ "1) Strongly Disagree", OPz_ScrnWRec == 2 ~ "2) Disagree",
    OPz_ScrnWRec == 3 ~ "3) Undecided", OPz_ScrnWRec == 4 ~ "4) Agree",
    OPz_ScrnWRec == 5 ~ "5) Strongly Agree")) %>% mutate(OPz_ScrnRecOthrs = case_when(OPz_ScrnRecOthrs ==
    1 ~ "1) Strongly Disagree", OPz_ScrnRecOthrs == 2 ~ "2) Disagree",
    OPz_ScrnRecOthrs == 3 ~ "3) Undecided", OPz_ScrnRecOthrs ==
        4 ~ "4) Agree", OPz_ScrnRecOthrs == 5 ~ "5) Strongly Agree")) %>%
    mutate(GNz_WRole = case_when(GNz_WRole == 1 ~ "1) Strongly Disagree",
        GNz_WRole == 2 ~ "2) Disagree", GNz_WRole == 3 ~ "3) Undecided",
        GNz_WRole == 4 ~ "4) Agree", GNz_WRole == 5 ~ "5) Strongly Agree")) %>%
    mutate(GNz_ManDecis = case_when(GNz_ManDecis == 1 ~ "1) Strongly Disagree",
        GNz_ManDecis == 2 ~ "2) Disagree", GNz_ManDecis == 3 ~
            "3) Undecided", GNz_ManDecis == 4 ~ "4) Agree", GNz_ManDecis ==
            5 ~ "5) Strongly Agree"))

TA_ScrnRec_Gendr <- tableby(SDz_Gender ~ fe(OPz_ScrnWRec, "countpct") +
    fe(OPz_ScrnRecOthrs, "countpct"), simulate.p.value = TRUE,
    data = ScrnRec_Gendr)

# OPz_ScrnWRec, OPz_ScrnRecOthrs, GNz_WRole, GNz_ManDecis,

labels(TA_ScrnRec_Gendr) <- c(OPz_ScrnWRec = "Other women that I know recommend the cervical cancer test",
    OPz_ScrnRecOthrs = "I would recommend that women get routine testing for cervical cancer")

summary(TA_ScrnRec_Gendr, title = "Arsenal Table 18Q04", pfootnote = TRUE)
```

```
##
## Table: Arsenal Table 18Q04
##
## |                                                                         | 0) Female (N=101) | 1) Male (N=57) | Total (N=158) |  p val
## |:------------------------------------------------------------------------|:-----------------:|:--------------:|:-------------:|:-------
```

```
## |**Other women that I know recommend the cervical cancer test**          |              |              |              | 0.822
## |   1) Strongly Disagree                                   |   2 (2.0%)   |   1 (1.8%)   |   3 (1.9%)   |
## |   2) Disagree                                            |   4 (4.0%)   |   3 (5.4%)   |   7 (4.5%)   |
## |   3) Undecided                                           |  30 (29.7%)  |  21 (37.5%)  |  51 (32.5%)  |
## |   4) Agree                                               |  43 (42.6%)  |  22 (39.3%)  |  65 (41.4%)  |
## |   5) Strongly Agree                                      |  22 (21.8%)  |  9 (16.1%)   |  31 (19.7%)  |
## |**I would recommend that women get routine testing for cervical cancer** |              |              |              | 0.412
## |   1) Strongly Disagree                                   |   3 (3.0%)   |   1 (1.8%)   |   4 (2.6%)   |
## |   2) Disagree                                            |   3 (3.0%)   |   0 (0.0%)   |   3 (1.9%)   |
## |   3) Undecided                                           |  11 (11.1%)  |   3 (5.3%)   |  14 (9.0%)   |
## |   4) Agree                                               |  33 (33.3%)  |  25 (43.9%)  |  58 (37.2%)  |
## |   5) Strongly Agree                                      |  49 (49.5%)  |  28 (49.1%)  |  77 (49.4%)  |
## 1. Fisher's Exact Test for Count Data with simulated p-value
##   (based on 2000 replicates)
```

```
# write2pdf(TA_ScrnRec_Gendr,
# here('Tables','T06_ScrnRec_Gendr.pdf'))
# write2word(TA_ScrnRec_Gendr,
# here('Tables','T06_ScrnRec_Gendr.doc'))
# write2html(TA_ScrnRec_Gendr,
# here('Tables','T06_ScrnRec_Gendr.html'))
```

#Supplement 1. Gender roles, perceptions of cancer stigma, cancer attitudes, and healthcare decision making by screening status

```
SocNorms_Scrnd <- Q1804dta_MSJoin %>% select(SDz_Gender, BEz_Screend,
    SDz_Educatn, OPz_ScrnWRec, OPz_ScrnRecOthrs, GNz_WRole, GNz_ManDecis,
    STz_CAComfNear, STz_CAAbnl, STz_CANeedsPriority, STz_CAPtFault,
    STz_CAFeelPity, STz_CADxFear, OPz_CAWorry, ATz_BnfComf, ATz_MyDxSlfKno,
    ATz_MyDxFamKno, ATz_FamDxMyKno, ATz_DxFate, DSz_HCSN, DSz_RespGen,
    DSz_RespPrtn, DSz_Smrt, DSz_Hnst, DSz_Thret, GNz_WHCDecisAll_01,
    GNz_WHCDecisAll_02, OPz_InfluenceWho_01, OPz_InfluenceWho_04,
    OPz_InfluenceWho_02, OPz_InfluenceWho_06, OPz_InfluenceWho_09,
    HSz_DecTrust_02, HSz_DecTrust_07, HSz_DecTrust_04, HSz_DecTrust_06,
    HSz_DecTrust_08, HSz_DecTrust_03, HSz_DecTrust_01) %>% mutate(SDz_Gender = case_when(SDz_Gender ==
    0 ~ "0) Female", SDz_Gender == 1 ~ "1) Male")) %>% mutate(BEz_Screend = case_when(BEz_Screend ==
    0 ~ "Not Screened", BEz_Screend == 1 ~ "Screened", BEz_Screend ==
    2 ~ "Screened x2", BEz_Screend == 99 ~ "Do Not Know / Not Sure")) %>%
    mutate(SDz_Educatn = case_when(SDz_Educatn == 0 ~ "0 Low Educ",
```

```
    SDz_Educatn == 1 ~ "0 Low Educ", SDz_Educatn == 2 ~ "1 sm Educ",
    SDz_Educatn == 3 ~ "1 sm Educ", SDz_Educatn == 4 ~ "High Educ",
    SDz_Educatn == 5 ~ "High Educ", SDz_Educatn == 99 ~ "Do Not Know / Not Sure")) %>%
mutate(STz_CAAbnl = case_when(STz_CAAbnl == 1 ~ "1) Strongly Disagree",
    STz_CAAbnl == 2 ~ "2) Disagree", STz_CAAbnl == 3 ~ "3) Undecided",
    STz_CAAbnl == 4 ~ "4) Agree", STz_CAAbnl == 5 ~ "5) Strongly Agree")) %>%
mutate(STz_CAComfNear = case_when(STz_CAComfNear == 1 ~ "1) Strongly Disagree",
    STz_CAComfNear == 2 ~ "2) Disagree", STz_CAComfNear ==
        3 ~ "3) Undecided", STz_CAComfNear == 4 ~ "4) Agree",
    STz_CAComfNear == 5 ~ "5) Strongly Agree")) %>% mutate(STz_CANeedsPriority = case_when(STz_CANeedsPriority ==
1 ~ "1) Strongly Disagree", STz_CANeedsPriority == 2 ~ "2) Disagree",
STz_CANeedsPriority == 3 ~ "3) Undecided", STz_CANeedsPriority ==
    4 ~ "4) Agree", STz_CANeedsPriority == 5 ~ "5) Strongly Agree")) %>%
mutate(STz_CAPtFault = case_when(STz_CAPtFault == 1 ~ "1) Strongly Disagree",
    STz_CAPtFault == 2 ~ "2) Disagree", STz_CAPtFault ==
        3 ~ "3) Undecided", STz_CAPtFault == 4 ~ "4) Agree",
    STz_CAPtFault == 5 ~ "5) Strongly Agree")) %>% mutate(STz_CAFeelPity = case_when(STz_CAFeelPity ==
1 ~ "1) Strongly Disagree", STz_CAFeelPity == 2 ~ "2) Disagree",
STz_CAFeelPity == 3 ~ "3) Undecided", STz_CAFeelPity == 4 ~
    "4) Agree", STz_CAFeelPity == 5 ~ "5) Strongly Agree")) %>%
mutate(STz_CADxFear = case_when(STz_CADxFear == 1 ~ "1) Strongly Disagree",
    STz_CADxFear == 2 ~ "2) Disagree", STz_CADxFear == 3 ~
        "3) Undecided", STz_CADxFear == 4 ~ "4) Agree", STz_CADxFear ==
        5 ~ "5) Strongly Agree")) %>% mutate(OPz_CAWorry = case_when(OPz_CAWorry ==
1 ~ "1) Strongly Disagree", OPz_CAWorry == 2 ~ "2) Disagree",
OPz_CAWorry == 3 ~ "3) Undecided", OPz_CAWorry == 4 ~ "4) Agree",
OPz_CAWorry == 5 ~ "5) Strongly Agree")) %>% mutate(DSz_RespGen = case_when(DSz_RespGen ==
1 ~ "1) Every day", DSz_RespGen == 2 ~ "2) Every week", DSz_RespGen ==
3 ~ "3) A few times per year", DSz_RespGen == 4 ~ "4) A few times in my life",
DSz_RespGen == 5 ~ "5) Never", DSz_RespGen == 99 ~ "Do Not Know / Not Sure")) %>%
mutate(DSz_RespPrtn = case_when(DSz_RespPrtn == 1 ~ "1) Every day",
    DSz_RespPrtn == 2 ~ "2) Every week", DSz_RespPrtn ==
        3 ~ "3) A few times per year", DSz_RespPrtn == 4 ~
        "4) A few times in my life", DSz_RespPrtn == 5 ~
        "5) Never", DSz_RespPrtn == 99 ~ "Do Not Know / Not Sure")) %>%
mutate(DSz_Smrt = case_when(DSz_Smrt == 1 ~ "1) Every day",
    DSz_Smrt == 2 ~ "2) Every week", DSz_Smrt == 3 ~ "3) A few times per year",
    DSz_Smrt == 4 ~ "4) A few times in my life", DSz_Smrt ==
```

```
        5 ~ "5) Never", DSz_Smrt == 99 ~ "Do Not Know / Not Sure")) %>%
    mutate(DSz_Hnst = case_when(DSz_Hnst == 1 ~ "1) Every day",
        DSz_Hnst == 2 ~ "2) Every week", DSz_Hnst == 3 ~ "3) A few times per year",
        DSz_Hnst == 4 ~ "4) A few times in my life", DSz_Hnst ==
            5 ~ "5) Never", DSz_Hnst == 99 ~ "Do Not Know / Not Sure")) %>%
    mutate(DSz_Thret = case_when(DSz_Thret == 1 ~ "1) Every day",
        DSz_Thret == 2 ~ "2) Every week", DSz_Thret == 3 ~ "3) A few times per year",
        DSz_Thret == 4 ~ "4) A few times in my life", DSz_Thret ==
            5 ~ "5) Never", DSz_Thret == 99 ~ "Do Not Know / Not Sure")) %>%
    mutate(ATz_BnfComf = case_when(ATz_BnfComf == 1 ~ "1) Strongly Disagree",
        ATz_BnfComf == 2 ~ "2) Disagree", ATz_BnfComf == 3 ~
            "3) Undecided", ATz_BnfComf == 4 ~ "4) Agree", ATz_BnfComf ==
            5 ~ "5) Strongly Agree")) %>% mutate(ATz_MyDxSlfKno = case_when(ATz_MyDxSlfKno ==
    1 ~ "1) Strongly Disagree", ATz_MyDxSlfKno == 2 ~ "2) Disagree",
    ATz_MyDxSlfKno == 3 ~ "3) Undecided", ATz_MyDxSlfKno == 4 ~
        "4) Agree", ATz_MyDxSlfKno == 5 ~ "5) Strongly Agree")) %>%
    mutate(ATz_MyDxFamKno = case_when(ATz_MyDxFamKno == 1 ~ "1) Strongly Disagree",
        ATz_MyDxFamKno == 2 ~ "2) Disagree", ATz_MyDxFamKno ==
            3 ~ "3) Undecided", ATz_MyDxFamKno == 4 ~ "4) Agree",
        ATz_MyDxFamKno == 5 ~ "5) Strongly Agree")) %>% mutate(ATz_FamDxMyKno = case_when(ATz_FamDxMyKno ==
    1 ~ "1) Strongly Disagree", ATz_FamDxMyKno == 2 ~ "2) Disagree",
    ATz_FamDxMyKno == 3 ~ "3) Undecided", ATz_FamDxMyKno == 4 ~
        "4) Agree", ATz_FamDxMyKno == 5 ~ "5) Strongly Agree")) %>%
    mutate(ATz_DxFate = case_when(ATz_DxFate == 1 ~ "1) Strongly Disagree",
        ATz_DxFate == 2 ~ "2) Disagree", ATz_DxFate == 3 ~ "3) Undecided",
        ATz_DxFate == 4 ~ "4) Agree", ATz_DxFate == 5 ~ "5) Strongly Agree")) %>%
    mutate(OPz_ScrnWRec = case_when(OPz_ScrnWRec == 1 ~ "1) Strongly Disagree",
        OPz_ScrnWRec == 2 ~ "2) Disagree", OPz_ScrnWRec == 3 ~
            "3) Undecided", OPz_ScrnWRec == 4 ~ "4) Agree", OPz_ScrnWRec ==
            5 ~ "5) Strongly Agree")) %>% mutate(OPz_ScrnRecOthrs = case_when(OPz_ScrnRecOthrs ==
    1 ~ "1) Strongly Disagree", OPz_ScrnRecOthrs == 2 ~ "2) Disagree",
    OPz_ScrnRecOthrs == 3 ~ "3) Undecided", OPz_ScrnRecOthrs ==
        4 ~ "4) Agree", OPz_ScrnRecOthrs == 5 ~ "5) Strongly Agree")) %>%
    mutate(GNz_WRole = case_when(GNz_WRole == 1 ~ "1) Strongly Disagree",
        GNz_WRole == 2 ~ "2) Disagree", GNz_WRole == 3 ~ "3) Undecided",
        GNz_WRole == 4 ~ "4) Agree", GNz_WRole == 5 ~ "5) Strongly Agree")) %>%
    mutate(GNz_ManDecis = case_when(GNz_ManDecis == 1 ~ "1) Strongly Disagree",
        GNz_ManDecis == 2 ~ "2) Disagree", GNz_ManDecis == 3 ~
```

```
            "3) Undecided", GNz_ManDecis == 4 ~ "4) Agree", GNz_ManDecis ==
            5 ~ "5) Strongly Agree"))

TA_SocNorms_Scrnd <- tableby(BEz_Screend ~ fe(OPz_ScrnWRec, "countpct") +
    fe(OPz_ScrnRecOthrs, "countpct") + fe(GNz_WRole, "countpct") +
    fe(GNz_ManDecis, "countpct") + fe(STz_CAComfNear, "countpct") +
    fe(STz_CAAbnl, "countpct") + fe(STz_CANeedsPriority, "countpct") +
    fe(STz_CAPtFault, "countpct") + fe(STz_CAFeelPity, "countpct") +
    fe(STz_CADxFear, "countpct") + fe(OPz_CAWorry, "countpct") +
    fe(ATz_BnfComf, "countpct") + fe(ATz_MyDxSlfKno, "countpct") +
    fe(ATz_MyDxFamKno, "countpct") + fe(ATz_FamDxMyKno, "countpct") +
    fe(ATz_DxFate, "countpct") + fe(DSz_HCSN, "countpct") + fe(DSz_RespGen,
    "countpct") + fe(DSz_RespPrtn, "countpct") + fe(DSz_Smrt,
    "countpct") + fe(DSz_Hnst, "countpct") + fe(DSz_Thret, "countpct") +
    fe(GNz_WHCDecisAll_01, "countpct") + fe(GNz_WHCDecisAll_02,
    "countpct") + fe(OPz_InfluenceWho_06, "countpct") + fe(OPz_InfluenceWho_04,
    "countpct") + fe(OPz_InfluenceWho_02, "countpct") + fe(OPz_InfluenceWho_09,
    "countpct") + fe(OPz_InfluenceWho_01, "countpct") + fe(HSz_DecTrust_02,
    "countpct") + fe(HSz_DecTrust_07, "countpct") + fe(HSz_DecTrust_04,
    "countpct") + fe(HSz_DecTrust_06, "countpct") + fe(HSz_DecTrust_08,
    "countpct") + fe(HSz_DecTrust_03, "countpct") + fe(HSz_DecTrust_01,
    "countpct"), simulate.p.value = TRUE, data = SocNorms_Scrnd)

labels(TA_SocNorms_Scrnd) <- c(STz_CAAbnl = "Once youve had cancer youre never normal again.",
    STz_CAComfNear = "I would not feel comfortable around someone with cancer.",
    STz_CANeedsPriority = "The health care needs of people with cancer should not be prioritized.",
    STz_CAPtFault = "If a person has cancer its probably their fault.",
    STz_CAFeelPity = " I would feel sorry for someone with cancer.",
    STz_CADxFear = "I feel that cancer is more frightening than most other diseases.",
    OPz_CAWorry = "Other women often state that they are worried about getting cancer.",
    ATz_BnfComf = "Cancer testing or treatment that is unpleasant is worth getting if it would help me to live longer",
    ATz_MyDxSlfKno = "If I had cancer, I would want to know that I have it",
    ATz_MyDxFamKno = "If I had cancer, I would want my family to know that I have it.",
    ATz_FamDxMyKno = "If someone else in my family had cancer, I would want to know that they have it.",
    ATz_DxFate = "Getting a serious disease like cancer is fate, there is nothing I can do to change fate",
    DSz_RespGen = "Feel treated with less courtesy or respect than others",
    DSz_RespPrtn = "Feel treated with less courtesy or respect by their spouse",
    DSz_Smrt = "Feel that others act as if they are not smart",
```

```
    DSz_Hnst = "Feel perceived as being dishonest", DSz_Thret = "Feel threatened by others",
    OPz_ScrnWRec = "Other women that I know recommend the cervical cancer test",
    OPz_ScrnRecOthrs = "I would recommend that women get routine testing for cervical cancer",
    GNz_WRole = "A womans most important role is to take care of her home and cook for her family",
    GNz_ManDecis = "A man should have the final word about decisions in his home",
    HSz_DecTrust_02 = "When making decisions about your healthcare, you most trust your spouse",
    HSz_DecTrust_07 = "When making decisions about your healthcare, you most trust your nurse",
    HSz_DecTrust_04 = "When making decisions about your healthcare, you most trust other family members",
    HSz_DecTrust_06 = "When making decisions about your healthcare, you most trust your physician",
    HSz_DecTrust_08 = "When making decisions about your healthcare, you most trust the community health worker",
    HSz_DecTrust_03 = "When making decisions about your healthcare, you most trust your children",
    HSz_DecTrust_01 = "When making decisions about your healthcare, you most trust yourself",
    GNz_WHCDecisAll_01 = "You have the final say at home regarding your healthcare decisions",
    GNz_WHCDecisAll_02 = "Your spouse has the final say at home regarding your healthcare decisions",
    OPz_InfluenceWho_04 = "The head of the household is most likely to influence your decision to get screened for cervical cancer",
    OPz_InfluenceWho_01 = "No one other than you will influence your decision to get screened for cervical cancer",
    OPz_InfluenceWho_02 = "Your spouse is most likely to influence your decision to get screened for cervical cancer",
    OPz_InfluenceWho_09 = "The nurse is most likely to influence your decision to get screened for cervical cancer",
    OPz_InfluenceWho_06 = "Other family members are most likely to influence your decision to get screened for cervical cancer",
    OPz_ScrnWRec = "Other women that I know recommend the cervical cancer test",
    OPz_ScrnRecOthrs = "I would recommend that women get routine testing for cervical cancer",
    GNz_WRole = "A womans most important role is to take care of her home and cook for her family",
    GNz_ManDecis = "A man should have the final word about decisions in his home")

summary(TA_SocNorms_Scrnd, title = "Arsenal Table 18Q04", pfootnote = TRUE)
```

```
##
## Table: Arsenal Table 18Q04
##
## |                                                                                                    | Not Screened (N=85) | Sc
## |:---------------------------------------------------------------------------------------------------|:-------------------:|:--
## |**Other women that I know recommend the cervical cancer test**                                      |                     |
## |   1) Strongly Disagree                                                              |      1 (1.2%)       |
## |   2) Disagree                                                                       |      2 (2.4%)       |
## |   3) Undecided                                                                      |     28 (32.9%)      |
## |   4) Agree                                                                          |     36 (42.4%)      |
## |   5) Strongly Agree                                                                 |     18 (21.2%)      |
## |**I would recommend that women get routine testing for cervical cancer**                            |                     |
```

```
## |   1) Strongly Disagree                                          |     2 (2.4%)     |
## |   2) Disagree                                                   |     3 (3.6%)     |
## |   3) Undecided                                                  |    11 (13.1%)    |
## |   4) Agree                                                      |    31 (36.9%)    |
## |   5) Strongly Agree                                             |    37 (44.0%)    |
## |**A womans most important role is to take care of her home and cook for her family** |            |
## |   1) Strongly Disagree                                          |     5 (6.0%)     |
## |   2) Disagree                                                   |    21 (25.0%)    |
## |   3) Undecided                                                  |     1 (1.2%)     |
## |   4) Agree                                                      |     7 (8.3%)     |
## |   5) Strongly Agree                                             |    50 (59.5%)    |
## |**A man should have the final word about decisions in his home**              |                  |
## |   1) Strongly Disagree                                          |     6 (7.1%)     |
## |   2) Disagree                                                   |    20 (23.5%)    |
## |   3) Undecided                                                  |     1 (1.2%)     |
## |   4) Agree                                                      |    12 (14.1%)    |
## |   5) Strongly Agree                                             |    46 (54.1%)    |
## |**I would not feel comfortable around someone with cancer.**                  |                  |
## |   1) Strongly Disagree                                          |     4 (4.7%)     |
## |   2) Disagree                                                   |    41 (48.2%)    |
## |   3) Undecided                                                  |     0 (0.0%)     |
## |   4) Agree                                                      |    19 (22.4%)    |
## |   5) Strongly Agree                                             |    21 (24.7%)    |
## |**Once youve had cancer youre never normal again.**                           |                  |
## |   1) Strongly Disagree                                          |     1 (1.2%)     |
## |   2) Disagree                                                   |     7 (8.2%)     |
## |   3) Undecided                                                  |     2 (2.4%)     |
## |   4) Agree                                                      |    29 (34.1%)    |
## |   5) Strongly Agree                                             |    46 (54.1%)    |
## |**The health care needs of people with cancer should not be prioritized.**    |                  |
## |   1) Strongly Disagree                                          |     6 (7.1%)     |
## |   2) Disagree                                                   |    20 (23.8%)    |
## |   3) Undecided                                                  |     3 (3.6%)     |
## |   4) Agree                                                      |    29 (34.5%)    |
## |   5) Strongly Agree                                             |    26 (31.0%)    |
## |**If a person has cancer its probably their fault.**                          |                  |
## |   1) Strongly Disagree                                          |     8 (9.4%)     |
## |   2) Disagree                                                   |    38 (44.7%)    |
## |   3) Undecided                                                  |     4 (4.7%)     |
```

```
## |   4) Agree                                                                         |      16 (18.8%)      |
## |   5) Strongly Agree                                                                |      19 (22.4%)      |
## |**I would feel sorry for someone with cancer.**                                                    |                      |
## |   1) Strongly Disagree                                                             |       1 (1.2%)       |
## |   2) Disagree                                                                      |       9 (10.6%)      |
## |   4) Agree                                                                         |      41 (48.2%)      |
## |   5) Strongly Agree                                                                |      34 (40.0%)      |
## |**I feel that cancer is more frightening than most other diseases.**                               |                      |
## |   2) Disagree                                                                      |       9 (10.7%)      |
## |   3) Undecided                                                                     |       4 (4.8%)       |
## |   4) Agree                                                                         |      30 (35.7%)      |
## |   5) Strongly Agree                                                                |      41 (48.8%)      |
## |**Other women often state that they are worried about getting cancer.**                            |                      |
## |   2) Disagree                                                                      |       2 (2.4%)       |
## |   3) Undecided                                                                     |      22 (25.9%)      |
## |   4) Agree                                                                         |      23 (27.1%)      |
## |   5) Strongly Agree                                                                |      38 (44.7%)      |
## |**Cancer testing or treatment that is unpleasant is worth getting if it would help me to live longer**      |                      |
## |   1) Strongly Disagree                                                             |       2 (2.4%)       |
## |   2) Disagree                                                                      |       7 (8.2%)       |
## |   3) Undecided                                                                     |       9 (10.6%)      |
## |   4) Agree                                                                         |      39 (45.9%)      |
## |   5) Strongly Agree                                                                |      28 (32.9%)      |
## |**If I had cancer, I would want to know that I have it**                                           |                      |
## |   1) Strongly Disagree                                                             |       1 (1.2%)       |
## |   2) Disagree                                                                      |       5 (5.9%)       |
## |   4) Agree                                                                         |      37 (43.5%)      |
## |   5) Strongly Agree                                                                |      42 (49.4%)      |
## |**If I had cancer, I would want my family to know that I have it.**                                |                      |
## |   1) Strongly Disagree                                                             |       7 (8.2%)       |
## |   2) Disagree                                                                      |      15 (17.6%)      |
## |   3) Undecided                                                                     |       3 (3.5%)       |
## |   4) Agree                                                                         |      36 (42.4%)      |
## |   5) Strongly Agree                                                                |      24 (28.2%)      |
## |**If someone else in my family had cancer, I would want to know that they have it.**               |                      |
## |   1) Strongly Disagree                                                             |       2 (2.4%)       |
## |   2) Disagree                                                                      |      23 (27.4%)      |
## |   4) Agree                                                                         |      34 (40.5%)      |
## |   5) Strongly Agree                                                                |      25 (29.8%)      |
```

```
## |**Getting a serious disease like cancer is fate, there is nothing I can do to change fate**        |                |
## |   1) Strongly Disagree                                                              |    16 (18.8%)  |
## |   2) Disagree                                                                       |    23 (27.1%)  |
## |   3) Undecided                                                                      |     6 (7.1%)   |
## |   4) Agree                                                                          |    29 (34.1%)  |
## |   5) Strongly Agree                                                                 |    11 (12.9%)  |
## |**DSz_HCSN**                                                                                        |                |
## |   0                                                                                 |    69 (83.1%)  |
## |   1                                                                                 |    13 (15.7%)  |
## |   2                                                                                 |     1 (1.2%)   |
## |**Feel treated with less courtesy or respect than others**                                          |                |
## |   1) Every day                                                                      |     9 (10.8%)  |
## |   2) Every week                                                                     |     2 (2.4%)   |
## |   3) A few times per year                                                           |     6 (7.2%)   |
## |   4) A few times in my life                                                         |    17 (20.5%)  |
## |   5) Never                                                                          |    49 (59.0%)  |
## |**Feel treated with less courtesy or respect by their spouse**                                      |                |
## |   1) Every day                                                                      |    11 (13.3%)  |
## |   2) Every week                                                                     |     4 (4.8%)   |
## |   3) A few times per year                                                           |    14 (16.9%)  |
## |   4) A few times in my life                                                         |    15 (18.1%)  |
## |   5) Never                                                                          |    39 (47.0%)  |
## |**Feel that others act as if they are not smart**                                                   |                |
## |   1) Every day                                                                      |     1 (1.2%)   |
## |   2) Every week                                                                     |     1 (1.2%)   |
## |   3) A few times per year                                                           |     4 (4.7%)   |
## |   4) A few times in my life                                                         |    14 (16.5%)  |
## |   5) Never                                                                          |    65 (76.5%)  |
## |**Feel perceived as being dishonest**                                                               |                |
## |   3) A few times per year                                                           |     1 (1.2%)   |
## |   4) A few times in my life                                                         |     9 (10.7%)  |
## |   5) Never                                                                          |    74 (88.1%)  |
## |**Feel threatened by others**                                                                       |                |
## |   1) Every day                                                                      |     1 (1.2%)   |
## |   3) A few times per year                                                           |     2 (2.4%)   |
## |   4) A few times in my life                                                         |    14 (16.9%)  |
## |   5) Never                                                                          |    66 (79.5%)  |
## |**You have the final say at home regarding your healthcare decisions**                              |                |
## |   No                                                                                |    73 (86.9%)  |
```

```
## |   Yes                                                                                   |      11 (13.1%)       |
## |**Your spouse has the final say at home regarding your healthcare decisions**                          |                      |
## |   No                                                                                    |      21 (25.0%)       |
## |   Yes                                                                                   |      63 (75.0%)       |
## |**Other family members are most likely to influence your decision to get screened for cervical cancer**   |                      |
## |   No                                                                                    |      76 (89.4%)       |
## |   Yes                                                                                   |       9 (10.6%)       |
## |**The head of the household is most likely to influence your decision to get screened for cervical cancer** |                      |
## |   No                                                                                    |      37 (43.5%)       |
## |   Yes                                                                                   |      48 (56.5%)       |
## |**Your spouse is most likely to influence your decision to get screened for cervical cancer**             |                      |
## |   No                                                                                    |      81 (95.3%)       |
## |   Yes                                                                                   |       4 (4.7%)        |
## |**The nurse is most likely to influence your decision to get screened for cervical cancer**               |                      |
## |   No                                                                                    |      84 (98.8%)       |
## |   Yes                                                                                   |       1 (1.2%)        |
## |**No one other than you will influence your decision to get screened for cervical cancer**                |                      |
## |   No                                                                                    |      56 (65.9%)       |
## |   Yes                                                                                   |      29 (34.1%)       |
## |**When making decisions about your healthcare, you most trust your spouse**                              |                      |
## |   No                                                                                    |      43 (50.6%)       |
## |   Yes                                                                                   |      42 (49.4%)       |
## |**When making decisions about your healthcare, you most trust your nurse**                               |                      |
## |   No                                                                                    |      70 (82.4%)       |
## |   Yes                                                                                   |      15 (17.6%)       |
## |**When making decisions about your healthcare, you most trust other family members**                     |                      |
## |   No                                                                                    |      71 (83.5%)       |
## |   Yes                                                                                   |      14 (16.5%)       |
## |**When making decisions about your healthcare, you most trust your physician**                           |                      |
## |   No                                                                                    |      84 (98.8%)       |
## |   Yes                                                                                   |       1 (1.2%)        |
## |**When making decisions about your healthcare, you most trust the community health worker**              |                      |
## |   No                                                                                    |      85 (100.0%)      |
## |**When making decisions about your healthcare, you most trust your children**                            |                      |
## |   No                                                                                    |      75 (88.2%)       |
## |   Yes                                                                                   |      10 (11.8%)       |
## |**When making decisions about your healthcare, you most trust yourself**                                 |                      |
## |   No                                                                                    |      78 (91.8%)       |
## |   Yes                                                                                   |       7 (8.2%)        |
```

```
## 1. Fisher's Exact Test for Count Data with simulated p-value
##    (based on 2000 replicates)
## 2. Fisher's Exact Test for Count Data
```

```
# write2pdf(TA_SocNorms_Scrnd,
# here('Tables','Sup_01_SocNorms_Scrnd.pdf'))
# write2word(TA_SocNorms_Scrnd,
# here('Tables','Sup_01_SocNorms_Scrnd.doc'))
# write2html(TA_SocNorms_Scrnd,
# here('Tables','Sup_01_SocNorms_Scrnd.html'))
```

#Supplement 2. Perception of gender roles by gender with effect modification by education

```
Power_Gendr_Ed <- Q1804dta_MSJoin %>% select(SDz_Gender, SDz_Educatn,
    OPz_ScrnWRec, OPz_ScrnRecOthrs, GNz_WRole, GNz_ManDecis,
    OPz_InfluenceWho_01, OPz_InfluenceWho_04, OPz_InfluenceWho_02,
    OPz_InfluenceWho_06, OPz_InfluenceWho_09, HSz_DecTrust_02,
    HSz_DecTrust_07, HSz_DecTrust_04, HSz_DecTrust_06, HSz_DecTrust_08,
    HSz_DecTrust_03, HSz_DecTrust_01, GNz_WHCDecisAll_01, GNz_WHCDecisAll_02) %>%
    mutate(SDz_Gender = case_when(SDz_Gender == 0 ~ "0) Female",
        SDz_Gender == 1 ~ "1) Male")) %>% mutate(OPz_ScrnWRec = case_when(OPz_ScrnWRec ==
    1 ~ "1) Strongly Disagree", OPz_ScrnWRec == 2 ~ "2) Disagree",
    OPz_ScrnWRec == 3 ~ "3) Undecided", OPz_ScrnWRec == 4 ~ "4) Agree",
    OPz_ScrnWRec == 5 ~ "5) Strongly Agree")) %>% mutate(OPz_ScrnRecOthrs = case_when(OPz_ScrnRecOthrs ==
    1 ~ "1) Strongly Disagree", OPz_ScrnRecOthrs == 2 ~ "2) Disagree",
    OPz_ScrnRecOthrs == 3 ~ "3) Undecided", OPz_ScrnRecOthrs ==
        4 ~ "4) Agree", OPz_ScrnRecOthrs == 5 ~ "5) Strongly Agree")) %>%
    mutate(GNz_WRole = case_when(GNz_WRole == 1 ~ "1) Strongly Disagree",
        GNz_WRole == 2 ~ "2) Disagree", GNz_WRole == 3 ~ "3) Undecided",
        GNz_WRole == 4 ~ "4) Agree", GNz_WRole == 5 ~ "5) Strongly Agree")) %>%
    mutate(GNz_ManDecis = case_when(GNz_ManDecis == 1 ~ "1) Strongly Disagree",
        GNz_ManDecis == 2 ~ "2) Disagree", GNz_ManDecis == 3 ~
            "3) Undecided", GNz_ManDecis == 4 ~ "4) Agree", GNz_ManDecis ==
            5 ~ "5) Strongly Agree")) %>% mutate(SDz_Educatn = case_when(SDz_Educatn ==
    0 ~ "0 Low Educ", SDz_Educatn == 1 ~ "0 Low Educ", SDz_Educatn ==
    2 ~ "1 Higher Educ", SDz_Educatn == 3 ~ "1 Higher Educ",
    SDz_Educatn == 4 ~ "1 Higher Educ", SDz_Educatn == 5 ~ "1 Higher Educ",
    SDz_Educatn == 99 ~ "Do Not Know / Not Sure"))
```

```r
TA_Power_Gendr_Ed <- tableby(interaction(SDz_Gender, SDz_Educatn) ~
    fe(GNz_WRole, "countpct") + fe(GNz_ManDecis, "countpct"),
    simulate.p.value = TRUE, data = Power_Gendr_Ed)

labels(TA_Power_Gendr_Ed) <- c(GNz_WRole = "A womans most important role is to take care of her home and cook for her family",
    GNz_ManDecis = "A man should have the final word about decisions in his home")

summary(TA_Power_Gendr_Ed, title = "Arsenal Table 18Q04", pfootnote = TRUE)
```

```
##
## Table: Arsenal Table 18Q04
##
## |                                                                                  | 0) Female.0 Low Educ (N=61) | 1) Male.0 Low Edu
## |:---------------------------------------------------------------------------------|:---------------------------:|:-----------------
## |**A womans most important role is to take care of her home and cook for her family** |                             |
## |   1) Strongly Disagree                                            |          4 (6.7%)           |         1 (3.8%)
## |   2) Disagree                                                     |          7 (11.7%)          |         1 (3.8%)
## |   3) Undecided                                                    |          2 (3.3%)           |         2 (7.7%)
## |   4) Agree                                                        |          6 (10.0%)          |        14 (53.8%)
## |   5) Strongly Agree                                               |         41 (68.3%)          |         8 (30.8%)
## |**A man should have the final word about decisions in his home**                  |                             |
## |   1) Strongly Disagree                                            |          7 (11.5%)          |         1 (3.8%)
## |   2) Disagree                                                     |          9 (14.8%)          |         0 (0.0%)
## |   3) Undecided                                                    |          1 (1.6%)           |         0 (0.0%)
## |   4) Agree                                                        |          9 (14.8%)          |        11 (42.3%)
## |   5) Strongly Agree                                               |         35 (57.4%)          |        14 (53.8%)
## 1. Fisher's Exact Test for Count Data with simulated p-value
##    (based on 2000 replicates)
```

```r
# write2pdf(TA_Power_Gendr_Ed,
# here('Tables','S02_Power_Gendr_Ed.pdf'))
# write2word(TA_Power_Gendr_Ed,
# here('Tables','S02_Power_Gendr_Ed.doc'))
# write2html(TA_Power_Gendr_Ed,
# here('Tables','S02_Power_Gendr_Ed.html'))
```

#Supplement 3. Adapted everyday discrimination scale by gender with effect modification by education.

```
Discr_Gendr_Ed <- Q1804dta_MSJoin %>% select(SDz_Gender, SDz_Educatn,
    DSz_RespGen, DSz_RespPrtn, DSz_Smrt, DSz_Hnst, DSz_Thret) %>%
    mutate(SDz_Gender = case_when(SDz_Gender == 0 ~ "0) Female",
        SDz_Gender == 1 ~ "1) Male")) %>% mutate(DSz_RespGen = case_when(DSz_RespGen ==
    1 ~ "1) Every day", DSz_RespGen == 2 ~ "2) Every week", DSz_RespGen ==
    3 ~ "3) A few times per year", DSz_RespGen == 4 ~ "4) A few times in my life",
    DSz_RespGen == 5 ~ "5) Never", DSz_RespGen == 99 ~ "Do Not Know / Not Sure")) %>%
    mutate(DSz_RespPrtn = case_when(DSz_RespPrtn == 1 ~ "1) Every day",
        DSz_RespPrtn == 2 ~ "2) Every week", DSz_RespPrtn ==
            3 ~ "3) A few times per year", DSz_RespPrtn == 4 ~
            "4) A few times in my life", DSz_RespPrtn == 5 ~
            "5) Never", DSz_RespPrtn == 99 ~ "Do Not Know / Not Sure")) %>%
    mutate(DSz_Smrt = case_when(DSz_Smrt == 1 ~ "1) Every day",
        DSz_Smrt == 2 ~ "2) Every week", DSz_Smrt == 3 ~ "3) A few times per year",
        DSz_Smrt == 4 ~ "4) A few times in my life", DSz_Smrt ==
            5 ~ "5) Never", DSz_Smrt == 99 ~ "Do Not Know / Not Sure")) %>%
    mutate(DSz_Hnst = case_when(DSz_Hnst == 1 ~ "1) Every day",
        DSz_Hnst == 2 ~ "2) Every week", DSz_Hnst == 3 ~ "3) A few times per year",
        DSz_Hnst == 4 ~ "4) A few times in my life", DSz_Hnst ==
            5 ~ "5) Never", DSz_Hnst == 99 ~ "Do Not Know / Not Sure")) %>%
    mutate(DSz_Thret = case_when(DSz_Thret == 1 ~ "1) Every day",
        DSz_Thret == 2 ~ "2) Every week", DSz_Thret == 3 ~ "3) A few times per year",
        DSz_Thret == 4 ~ "4) A few times in my life", DSz_Thret ==
            5 ~ "5) Never", DSz_Thret == 99 ~ "Do Not Know / Not Sure")) %>%
    mutate(SDz_Educatn = case_when(SDz_Educatn == 0 ~ "0 Low Educ",
        SDz_Educatn == 1 ~ "0 Low Educ", SDz_Educatn == 2 ~ "1 Higher Educ",
        SDz_Educatn == 3 ~ "1 Higher Educ", SDz_Educatn == 4 ~
            "1 Higher Educ", SDz_Educatn == 5 ~ "1 Higher Educ",
        SDz_Educatn == 99 ~ "Do Not Know / Not Sure"))

TA_Discr_Gendr_Ed <- tableby(interaction(SDz_Gender, SDz_Educatn) ~
    fe(DSz_RespGen, "countpct") + fe(DSz_RespPrtn, "countpct") +
        fe(DSz_Smrt, "countpct") + fe(DSz_Hnst, "countpct") +
        fe(DSz_Thret, "countpct"), simulate.p.value = TRUE, data = Discr_Gendr_Ed)

labels(TA_Discr_Gendr_Ed) <- c(DSz_RespGen = "Feel treated with less courtesy or respect than others",
    DSz_RespPrtn = "Feel treated with less courtesy or respect by their spouse",
    DSz_Smrt = "Feel that others act as if they are not smart",
```

```r
    DSz_Hnst = "Feel perceived as being dishonest", DSz_Thret = "Feel threatened by others")

summary(TA_Discr_Gendr_Ed, title = "Arsenal Table 18Q04", pfootnote = TRUE)
```

```
##
## Table: Arsenal Table 18Q04
##
## |                                                                | 0) Female.0 Low Educ (N=61) | 1) Male.0 Low Educ (N=26) | 0) Female.1
## |:---------------------------------------------------------------|:---------------------------:|:-------------------------:|:-----------
## |**Feel treated with less courtesy or respect than others**      |                             |                           |
## |   1) Every day                                  |         6 (10.2%)           |          0 (0.0%)         |
## |   2) Every week                                 |          1 (1.7%)           |          0 (0.0%)         |
## |   3) A few times per year                       |         6 (10.2%)           |          0 (0.0%)         |
## |   4) A few times in my life                     |         7 (11.9%)           |         3 (12.5%)         |          1
## |   5) Never                                      |        39 (66.1%)           |        21 (87.5%)         |          2
## |**Feel treated with less courtesy or respect by their spouse** |                             |                           |
## |   1) Every day                                  |        10 (16.9%)           |          0 (0.0%)         |          4
## |   2) Every week                                 |          4 (6.8%)           |          0 (0.0%)         |
## |   3) A few times per year                       |         9 (15.3%)           |          1 (4.5%)         |          6
## |   4) A few times in my life                     |         7 (11.9%)           |         5 (22.7%)         |          1
## |   5) Never                                      |        29 (49.2%)           |        16 (72.7%)         |          1
## |**Feel that others act as if they are not smart**               |                             |                           |
## |   1) Every day                                  |          1 (1.6%)           |          0 (0.0%)         |
## |   2) Every week                                 |          0 (0.0%)           |          0 (0.0%)         |
## |   3) A few times per year                       |          3 (4.9%)           |         3 (12.5%)         |
## |   4) A few times in my life                     |          3 (4.9%)           |         6 (25.0%)         |          1
## |   5) Never                                      |        54 (88.5%)           |        15 (62.5%)         |          2
## |**Feel perceived as being dishonest**                           |                             |                           |
## |   3) A few times per year                       |          1 (1.7%)           |          0 (0.0%)         |
## |   4) A few times in my life                     |          2 (3.3%)           |         5 (25.0%)         |          9
## |   5) Never                                      |        57 (95.0%)           |        15 (75.0%)         |          3
## |**Feel threatened by others**                                   |                             |                           |
## |   1) Every day                                  |          1 (1.7%)           |          0 (0.0%)         |
## |   3) A few times per year                       |          3 (5.1%)           |          1 (4.8%)         |
## |   4) A few times in my life                     |         6 (10.2%)           |         4 (19.0%)         |          1
## |   5) Never                                      |        49 (83.1%)           |        16 (76.2%)         |          3
## 1. Fisher's Exact Test for Count Data with simulated p-value
##    (based on 2000 replicates)
```

```
# write2pdf(TA_Discr_Gendr_Ed,
# here('Tables','S03_Discr_Gendr_Ed.pdf'))
# write2word(TA_Discr_Gendr_Ed,
# here('Tables','S03_Discr_Gendr_Ed.doc'))
# write2html(TA_Discr_Gendr_Ed,
# here('Tables','S03_Discr_Gendr_Ed.html'))
```

#Supplement 4. Adapted cancer stigma scale by gender with effect modification by education.

```
Stgma_Gendr_Ed <- Q1804dta_MSJoin %>% select(SDz_Gender, SDz_Educatn,
    STz_CAAbnl, STz_CAComfNear, STz_CANeedsPriority, STz_CAPtFault,
    STz_CAFeelPity, STz_CADxFear, OPz_CAWorry) %>% mutate(SDz_Gender = case_when(SDz_Gender ==
    0 ~ "0) Female", SDz_Gender == 1 ~ "1) Male")) %>% mutate(STz_CAAbnl = case_when(STz_CAAbnl ==
    1 ~ "1) Strongly Disagree", STz_CAAbnl == 2 ~ "2) Disagree",
    STz_CAAbnl == 3 ~ "3) Undecided", STz_CAAbnl == 4 ~ "4) Agree",
    STz_CAAbnl == 5 ~ "5) Strongly Agree")) %>% mutate(STz_CAComfNear = case_when(STz_CAComfNear ==
    1 ~ "1) Strongly Disagree", STz_CAComfNear == 2 ~ "2) Disagree",
    STz_CAComfNear == 3 ~ "3) Undecided", STz_CAComfNear == 4 ~
        "4) Agree", STz_CAComfNear == 5 ~ "5) Strongly Agree")) %>%
    mutate(STz_CANeedsPriority = case_when(STz_CANeedsPriority ==
        1 ~ "1) Strongly Disagree", STz_CANeedsPriority == 2 ~
        "2) Disagree", STz_CANeedsPriority == 3 ~ "3) Undecided",
        STz_CANeedsPriority == 4 ~ "4) Agree", STz_CANeedsPriority ==
            5 ~ "5) Strongly Agree")) %>% mutate(STz_CAPtFault = case_when(STz_CAPtFault ==
    1 ~ "1) Strongly Disagree", STz_CAPtFault == 2 ~ "2) Disagree",
    STz_CAPtFault == 3 ~ "3) Undecided", STz_CAPtFault == 4 ~
        "4) Agree", STz_CAPtFault == 5 ~ "5) Strongly Agree")) %>%
    mutate(STz_CAFeelPity = case_when(STz_CAFeelPity == 1 ~ "1) Strongly Disagree",
        STz_CAFeelPity == 2 ~ "2) Disagree", STz_CAFeelPity ==
            3 ~ "3) Undecided", STz_CAFeelPity == 4 ~ "4) Agree",
        STz_CAFeelPity == 5 ~ "5) Strongly Agree")) %>% mutate(STz_CADxFear = case_when(STz_CADxFear ==
    1 ~ "1) Strongly Disagree", STz_CADxFear == 2 ~ "2) Disagree",
    STz_CADxFear == 3 ~ "3) Undecided", STz_CADxFear == 4 ~ "4) Agree",
    STz_CADxFear == 5 ~ "5) Strongly Agree")) %>% mutate(OPz_CAWorry = case_when(OPz_CAWorry ==
    1 ~ "1) Strongly Disagree", OPz_CAWorry == 2 ~ "2) Disagree",
    OPz_CAWorry == 3 ~ "3) Undecided", OPz_CAWorry == 4 ~ "4) Agree",
    OPz_CAWorry == 5 ~ "5) Strongly Agree")) %>% mutate(SDz_Educatn = case_when(SDz_Educatn ==
    0 ~ "0 Low Educ", SDz_Educatn == 1 ~ "0 Low Educ", SDz_Educatn ==
```

```r
    2 ~ "1 Higher Educ", SDz_Educatn == 3 ~ "1 Higher Educ",
    SDz_Educatn == 4 ~ "1 Higher Educ", SDz_Educatn == 5 ~ "1 Higher Educ",
    SDz_Educatn == 99 ~ "Do Not Know / Not Sure"))

TA_Stgma_Gendr_Ed <- tableby(interaction(SDz_Gender, SDz_Educatn) ~
    fe(STz_CAComfNear, "countpct") + fe(STz_CAAbnl, "countpct") +
        fe(STz_CANeedsPriority, "countpct") + fe(STz_CAPtFault,
        "countpct") + fe(STz_CAFeelPity, "countpct") + fe(STz_CADxFear,
        "countpct") + fe(OPz_CAWorry, "countpct"), simulate.p.value = TRUE,
    data = Stgma_Gendr_Ed)

labels(TA_Stgma_Gendr_Ed) <- c(STz_CAAbnl = "Once youve had cancer youre never normal again.",
    STz_CAComfNear = "I would not feel comfortable around someone with cancer.",
    STz_CANeedsPriority = "The health care needs of people with cancer should not be prioritized.",
    STz_CAPtFault = "If a person has cancer its probably their fault.",
    STz_CAFeelPity = " I would feel sorry for someone with cancer.",
    STz_CADxFear = "I feel that cancer is more frightening than most other diseases.",
    OPz_CAWorry = "Other women often state that they are worried about getting cancer.")

summary(TA_Stgma_Gendr_Ed, title = "Arsenal Table 18Q04", pfootnote = TRUE)
```

```
##
## Table: Arsenal Table 18Q04
##
## |                                                                          | 0) Female.0 Low Educ (N=61) | 1) Male.0 Low Educ (N=26) |
## |:-------------------------------------------------------------------------|:---------------------------:|:-------------------------:|
## |**I would not feel comfortable around someone with cancer.**              |                             |                           |
## |   1) Strongly Disagree                                    |         7 (11.5%)           |        2 (7.7%)           |
## |   2) Disagree                                             |        21 (34.4%)           |       20 (76.9%)          |
## |   3) Undecided                                            |         0 (0.0%)            |        2 (7.7%)           |
## |   4) Agree                                                |        13 (21.3%)           |        2 (7.7%)           |
## |   5) Strongly Agree                                       |        20 (32.8%)           |        0 (0.0%)           |
## |**Once youve had cancer youre never normal again.**                       |                             |                           |
## |   1) Strongly Disagree                                    |         1 (1.6%)            |        1 (3.8%)           |
## |   2) Disagree                                             |         3 (4.9%)            |        6 (23.1%)          |
## |   3) Undecided                                            |         1 (1.6%)            |        6 (23.1%)          |
## |   4) Agree                                                |        18 (29.5%)           |       10 (38.5%)          |
## |   5) Strongly Agree                                       |        38 (62.3%)           |        3 (11.5%)          |
```

```
## |**The health care needs of people with cancer should not be prioritized.** |                    |                    |
## |   1) Strongly Disagree                                       |          7 (11.5%) |          5 (19.2%) |
## |   2) Disagree                                                |         14 (23.0%) |         18 (69.2%) |
## |   3) Undecided                                               |          3 (4.9%)  |          1 (3.8%)  |
## |   4) Agree                                                   |         17 (27.9%) |          2 (7.7%)  |
## |   5) Strongly Agree                                          |         20 (32.8%) |          0 (0.0%)  |
## |**If a person has cancer its probably their fault.**                         |                    |                    |
## |   1) Strongly Disagree                                       |          8 (13.1%) |          4 (15.4%) |
## |   2) Disagree                                                |         17 (27.9%) |         13 (50.0%) |
## |   3) Undecided                                               |          3 (4.9%)  |          8 (30.8%) |
## |   4) Agree                                                   |         13 (21.3%) |          1 (3.8%)  |
## |   5) Strongly Agree                                          |         20 (32.8%) |          0 (0.0%)  |
## |**I would feel sorry for someone with cancer.**                              |                    |                    |
## |   1) Strongly Disagree                                       |          0 (0.0%)  |          2 (7.7%)  |
## |   2) Disagree                                                |          9 (14.8%) |          1 (3.8%)  |
## |   4) Agree                                                   |         28 (45.9%) |         12 (46.2%) |
## |   5) Strongly Agree                                          |         24 (39.3%) |         11 (42.3%) |
## |**I feel that cancer is more frightening than most other diseases.**         |                    |                    |
## |   1) Strongly Disagree                                       |          0 (0.0%)  |          1 (3.8%)  |
## |   2) Disagree                                                |          5 (8.2%)  |          4 (15.4%) |
## |   3) Undecided                                               |          2 (3.3%)  |          3 (11.5%) |
## |   4) Agree                                                   |         24 (39.3%) |         11 (42.3%) |
## |   5) Strongly Agree                                          |         30 (49.2%) |          7 (26.9%) |
## |**Other women often state that they are worried about getting cancer.**      |                    |                    |
## |   1) Strongly Disagree                                       |          0 (0.0%)  |          1 (3.8%)  |
## |   2) Disagree                                                |          2 (3.3%)  |          0 (0.0%)  |
## |   3) Undecided                                               |         11 (18.0%) |         15 (57.7%) |
## |   4) Agree                                                   |         12 (19.7%) |          8 (30.8%) |
## |   5) Strongly Agree                                          |         36 (59.0%) |          2 (7.7%)  |
## 1. Fisher's Exact Test for Count Data with simulated p-value
##   (based on 2000 replicates)
```

```
# write2pdf(TA_Stgma_Gendr_Ed,
# here('Tables','S04_Stgma_Gendr_Ed.pdf'))
# write2word(TA_Stgma_Gendr_Ed,
# here('Tables','S04_Stgma_Gendr_Ed.doc'))
# write2html(TA_Stgma_Gendr_Ed,
# here('Tables','S04_Stgma_Gendr_Ed.html'))
```

#Supplement 5. Cancer Attitudes by gender with effect modification by education.

```
Attd_Gendr_Ed <- Q1804dta_MSJoin %>% select(SDz_Gender, SDz_Educatn,
    BEz_Screend, ATz_BnfComf, ATz_MyDxSlfKno, ATz_MyDxFamKno,
    ATz_FamDxMyKno, ATz_DxFate) %>% mutate(SDz_Gender = case_when(SDz_Gender ==
    0 ~ "0) Female", SDz_Gender == 1 ~ "1) Male")) %>% mutate(ATz_BnfComf = case_when(ATz_BnfComf ==
    1 ~ "1) Strongly Disagree", ATz_BnfComf == 2 ~ "2) Disagree",
    ATz_BnfComf == 3 ~ "3) Undecided", ATz_BnfComf == 4 ~ "4) Agree",
    ATz_BnfComf == 5 ~ "5) Strongly Agree")) %>% mutate(ATz_MyDxSlfKno = case_when(ATz_MyDxSlfKno ==
    1 ~ "1) Strongly Disagree", ATz_MyDxSlfKno == 2 ~ "2) Disagree",
    ATz_MyDxSlfKno == 3 ~ "3) Undecided", ATz_MyDxSlfKno == 4 ~
        "4) Agree", ATz_MyDxSlfKno == 5 ~ "5) Strongly Agree")) %>%
    mutate(ATz_MyDxFamKno = case_when(ATz_MyDxFamKno == 1 ~ "1) Strongly Disagree",
        ATz_MyDxFamKno == 2 ~ "2) Disagree", ATz_MyDxFamKno ==
            3 ~ "3) Undecided", ATz_MyDxFamKno == 4 ~ "4) Agree",
        ATz_MyDxFamKno == 5 ~ "5) Strongly Agree")) %>% mutate(ATz_FamDxMyKno = case_when(ATz_FamDxMyKno ==
    1 ~ "1) Strongly Disagree", ATz_FamDxMyKno == 2 ~ "2) Disagree",
    ATz_FamDxMyKno == 3 ~ "3) Undecided", ATz_FamDxMyKno == 4 ~
        "4) Agree", ATz_FamDxMyKno == 5 ~ "5) Strongly Agree")) %>%
    mutate(ATz_DxFate = case_when(ATz_DxFate == 1 ~ "1) Strongly Disagree",
        ATz_DxFate == 2 ~ "2) Disagree", ATz_DxFate == 3 ~ "3) Undecided",
        ATz_DxFate == 4 ~ "4) Agree", ATz_DxFate == 5 ~ "5) Strongly Agree")) %>%
    mutate(SDz_Educatn = case_when(SDz_Educatn == 0 ~ "0 Low Educ",
        SDz_Educatn == 1 ~ "0 Low Educ", SDz_Educatn == 2 ~ "1 Higher Educ",
        SDz_Educatn == 3 ~ "1 Higher Educ", SDz_Educatn == 4 ~
            "1 Higher Educ", SDz_Educatn == 5 ~ "1 Higher Educ",
        SDz_Educatn == 99 ~ "Do Not Know / Not Sure"))

TA_Attd_Gendr_Ed <- tableby(interaction(SDz_Gender, SDz_Educatn) ~
    fe(ATz_BnfComf, "countpct") + fe(ATz_MyDxSlfKno, "countpct") +
        fe(ATz_MyDxFamKno, "countpct") + fe(ATz_FamDxMyKno, "countpct") +
        fe(ATz_DxFate, "countpct"), simulate.p.value = TRUE,
    data = Attd_Gendr_Ed)

labels(TA_Attd_Gendr_Ed) <- c(ATz_BnfComf = "Cancer testing or treatment that is unpleasant is worth getting if it would help me to live l
    ATz_MyDxSlfKno = "If I had cancer, I would want to know that I have it",
    ATz_MyDxFamKno = "If I had cancer, I would want my family to know that I have it.",
    ATz_FamDxMyKno = "If someone else in my family had cancer, I would want to know that they have it.",
    ATz_DxFate = "Getting a serious disease like cancer is fate, there is nothing I can do to change fate")
```

```r
summary(TA_Attd_Gendr_Ed, title = "Arsenal Table 18Q04", pfootnote = TRUE)
```

```
##
## Table: Arsenal Table 18Q04
##
## |                                                                                           | 0) Female.0 Low Educ (N=61) |
## |:------------------------------------------------------------------------------------------|:---------------------------:|
## |**Cancer testing or treatment that is unpleasant is worth getting if it would help me to live longer** |               |
## |   1) Strongly Disagree                                                     |          2 (3.3%)           |
## |   2) Disagree                                                              |          2 (3.3%)           |
## |   3) Undecided                                                             |          4 (6.6%)           |
## |   4) Agree                                                                 |         25 (41.0%)          |
## |   5) Strongly Agree                                                        |         28 (45.9%)          |
## |**If I had cancer, I would want to know that I have it**                                   |                             |
## |   1) Strongly Disagree                                                     |          0 (0.0%)           |
## |   2) Disagree                                                              |          2 (3.3%)           |
## |   3) Undecided                                                             |          0 (0.0%)           |
## |   4) Agree                                                                 |         23 (38.3%)          |
## |   5) Strongly Agree                                                        |         35 (58.3%)          |
## |**If I had cancer, I would want my family to know that I have it.**                        |                             |
## |   1) Strongly Disagree                                                     |          9 (14.8%)          |
## |   2) Disagree                                                              |          8 (13.1%)          |
## |   3) Undecided                                                             |          2 (3.3%)           |
## |   4) Agree                                                                 |         24 (39.3%)          |
## |   5) Strongly Agree                                                        |         18 (29.5%)          |
## |**If someone else in my family had cancer, I would want to know that they have it.**        |                             |
## |   1) Strongly Disagree                                                     |          3 (5.0%)           |
## |   2) Disagree                                                              |         15 (25.0%)          |
## |   4) Agree                                                                 |         19 (31.7%)          |
## |   5) Strongly Agree                                                        |         23 (38.3%)          |
## |**Getting a serious disease like cancer is fate, there is nothing I can do to change fate** |                             |
## |   1) Strongly Disagree                                                     |         21 (34.4%)          |
## |   2) Disagree                                                              |         11 (18.0%)          |
## |   3) Undecided                                                             |          3 (4.9%)           |
## |   4) Agree                                                                 |         21 (34.4%)          |
## |   5) Strongly Agree                                                        |          5 (8.2%)           |
## 1. Fisher's Exact Test for Count Data with simulated p-value
##    (based on 2000 replicates)
```

```
# write2pdf(TA_Attd_Gendr_Ed,
# here('Tables','S05_Attd_Gendr_Ed.pdf'))
# write2word(TA_Attd_Gendr_Ed,
# here('Tables','S05_Attd_Gendr_Ed.doc'))
# write2html(TA_Attd_Gendr_Ed,
# here('Tables','S05_Attd_Gendr_Ed.html'))
```

#Supplement 6. Cervical cancer screening recommendation by gender with effect modification by education.

```
ScrnRec_Gendr_Ed <- Q1804dta_MSJoin %>% select(SDz_Gender, SDz_Educatn,
    OPz_ScrnWRec, OPz_ScrnRecOthrs, GNz_WRole, GNz_ManDecis,
    OPz_InfluenceWho_01, OPz_InfluenceWho_04, OPz_InfluenceWho_02,
    OPz_InfluenceWho_06, OPz_InfluenceWho_09, HSz_DecTrust_02,
    HSz_DecTrust_07, HSz_DecTrust_04, HSz_DecTrust_06, HSz_DecTrust_08,
    HSz_DecTrust_03, HSz_DecTrust_01, GNz_WHCDecisAll_01, GNz_WHCDecisAll_02) %>%
    mutate(SDz_Gender = case_when(SDz_Gender == 0 ~ "0) Female",
        SDz_Gender == 1 ~ "1) Male")) %>% mutate(OPz_ScrnWRec = case_when(OPz_ScrnWRec ==
    1 ~ "1) Strongly Disagree", OPz_ScrnWRec == 2 ~ "2) Disagree",
    OPz_ScrnWRec == 3 ~ "3) Undecided", OPz_ScrnWRec == 4 ~ "4) Agree",
    OPz_ScrnWRec == 5 ~ "5) Strongly Agree")) %>% mutate(OPz_ScrnRecOthrs = case_when(OPz_ScrnRecOthrs ==
    1 ~ "1) Strongly Disagree", OPz_ScrnRecOthrs == 2 ~ "2) Disagree",
    OPz_ScrnRecOthrs == 3 ~ "3) Undecided", OPz_ScrnRecOthrs ==
        4 ~ "4) Agree", OPz_ScrnRecOthrs == 5 ~ "5) Strongly Agree")) %>%
    mutate(GNz_WRole = case_when(GNz_WRole == 1 ~ "1) Strongly Disagree",
        GNz_WRole == 2 ~ "2) Disagree", GNz_WRole == 3 ~ "3) Undecided",
        GNz_WRole == 4 ~ "4) Agree", GNz_WRole == 5 ~ "5) Strongly Agree")) %>%
    mutate(GNz_ManDecis = case_when(GNz_ManDecis == 1 ~ "1) Strongly Disagree",
        GNz_ManDecis == 2 ~ "2) Disagree", GNz_ManDecis == 3 ~
            "3) Undecided", GNz_ManDecis == 4 ~ "4) Agree", GNz_ManDecis ==
            5 ~ "5) Strongly Agree")) %>% mutate(SDz_Educatn = case_when(SDz_Educatn ==
    0 ~ "0 Low Educ", SDz_Educatn == 1 ~ "0 Low Educ", SDz_Educatn ==
    2 ~ "1 Higher Educ", SDz_Educatn == 3 ~ "1 Higher Educ",
    SDz_Educatn == 4 ~ "1 Higher Educ", SDz_Educatn == 5 ~ "1 Higher Educ",
    SDz_Educatn == 99 ~ "Do Not Know / Not Sure"))

TA_ScrnRec_Gendr_Ed <- tableby(interaction(SDz_Gender, SDz_Educatn) ~
    fe(OPz_ScrnWRec, "countpct") + fe(OPz_ScrnRecOthrs, "countpct"),
    simulate.p.value = TRUE, data = ScrnRec_Gendr_Ed)
```

```r
# OPz_ScrnWRec, OPz_ScrnRecOthrs, GNz_WRole, GNz_ManDecis,

labels(TA_ScrnRec_Gendr_Ed) <- c(OPz_ScrnWRec = "Other women that I know recommend the cervical cancer test",
    OPz_ScrnRecOthrs = "I would recommend that women get routine testing for cervical cancer")

summary(TA_ScrnRec_Gendr_Ed, title = "Arsenal Table 18Q04", pfootnote = TRUE)
```

```
##
## Table: Arsenal Table 18Q04
##
## |                                                                        | 0) Female.0 Low Educ (N=61) | 1) Male.0 Low Educ (N=26) | (
## |:-----------------------------------------------------------------------|:---------------------------:|:-------------------------:|:-
## |**Other women that I know recommend the cervical cancer test**          |                             |                           |
## |   1) Strongly Disagree                                  |          1 (1.6%)           |         1 (3.8%)          |
## |   2) Disagree                                           |          4 (6.6%)           |         1 (3.8%)          |
## |   3) Undecided                                          |         13 (21.3%)          |         8 (30.8%)         |
## |   4) Agree                                              |         26 (42.6%)          |         9 (34.6%)         |
## |   5) Strongly Agree                                     |         17 (27.9%)          |         7 (26.9%)         |
## |**I would recommend that women get routine testing for cervical cancer** |                             |                           |
## |   1) Strongly Disagree                                  |          2 (3.3%)           |         1 (3.8%)          |
## |   2) Disagree                                           |          2 (3.3%)           |         0 (0.0%)          |
## |   3) Undecided                                          |          5 (8.3%)           |         1 (3.8%)          |
## |   4) Agree                                              |         18 (30.0%)          |        11 (42.3%)         |
## |   5) Strongly Agree                                     |         33 (55.0%)          |        13 (50.0%)         |
## 1. Fisher's Exact Test for Count Data with simulated p-value
##    (based on 2000 replicates)
```

```r
# write2pdf(TA_ScrnRec_Gendr_Ed,
# here('Tables','S06_ScrnRec_Gendr_Ed.pdf'))
# write2word(TA_ScrnRec_Gendr_Ed,
# here('Tables','S06_ScrnRec_Gendr_Ed.doc'))
# write2html(TA_ScrnRec_Gendr_Ed,
# here('Tables','S06_ScrnRec_Gendr_Ed.html'))
```
