# Supplementary material for: Role of gender in perspectives of discrimination, stigma, and attitudes relative to cervical cancer in rural Sénégal
Source: PLoS One. 2020 Apr 28;15(4):e0232291. doi: 10.1371/journal.pone.0232291 (PMC7188246; doi:10.1371/journal.pone.0232291)
Supplement: S1 Questionnaire — (DOCX) [file pone.0232291.s004.docx]

***Instructions to the interviewer***:

Leave box empty - For administration use only

**Participant # _______________**

**DCE#: DCI K011601.__ __ __**

**Data Entry Completion Date: ______**

- Please clearly circle the number next to the person’s response.
- Except where indicated, please circle only one answer for each question.
- Except where indicated (special instructions for questions are in CAPITAL letters), read the question and the responses to the respondent.
- Words in CAPITAL letters are NOT read, while words in lowercase letters ARE read to the respondent.

**Refusing to answer, Uncomfortable with Question, Misunderstanding**

- If the respondent refuses to answer a question, please circle an (R)* on the right side of the question response area.
- If the respondent appears uncomfortable with the question, please circle a (U)* on the right side of the question response area.
- If the respondent seems to misunderstand or misinterpret the question, please circle an (M)*on the right side of the question response area.
- You may circle multiple letters for any given question.

**PLEASE FILL IN THE FOLLOWING INFORMATION FOR ALL INTERVIEWS:**

| Admin 02 | INTERVIEWER |  |
| --- | --- | --- |
| Admin 03 | DATE | _____/ _____ / 20___ ( DD / MM / YYYY ) |
| Admin 04 | TIME BEGIN |  |
| Admin 05 | TIME END |  |
| Admin 06 | CLUSTER # | 1. SALEMATA DISTRICT 2. SARAYA DISTRICT 3. KEDOUGOU DISTRICT |
| Admin 07 | COMMUNITY # | SALEMATA DISTRICT 11 - Salemata 12 - Dar Salaam 13 - Dakately  SARAYA DISTRICT 21 - Saraya 22 - Nafadji 23 - Khossanto KEDOUGOU DISTRICT 31 - Kedougou - Dalaba  32 - Bandifassi 33 - Dindefello |
| Admin 08 | HOUSEHOLD # OR  CARE GROUP MEMBER # |  |

| **1** | GENDER | 0) FEMALE |  |
| --- | --- | --- | --- |
| **2** | What is your age? |  |  |
| **3** | What language(s) do you prefer to speak? Do you prefer…   (Tell me only the language(s) that you prefer, regardless of competency)  (CIRCLE ALL THAT APPLY) | MULTIPLE ANSWERS POSSIBLE  1) Malinke  2) Pulaar  3) Basari  4) Bedick  5) Serer  6) Wolof  7) French  88) Other  99) DON’T KNOW / NOT SURE | R   U   M |
| **4** | IF "OTHER,"   Please specify. |  |  |
| **5** | Has a healthcare provider ever tested you for cervical cancer? | 0) No, I have never been screened  1) Yes, one time only  2) Yes, more than one time  99) DON'T KNOW / NOT SURE | R   U   M |
| **6** | What is the highest level of education that you have completed? | 0) None 1) Quranic School 2) Primary education 3) Secondary school, up to two years 4) Secondary school, more than two years 5) Some university education 99) DON'T KNOW / NOT SURE | R   U   M |
| **7** | What is your marital status? | 1) Single 2) Married (monogamous household) 3) Married (polygamous household)  4) Living as married / cohabitating 5) Divorced or Separated 6) Widowed 99) DON'T KNOW / NOT SURE | R   U   M |
| **8** | Who do you most trust in helping you make decisions about your own health care? |  | R   U   M |
| **9** | In the following questions, I am going to ask you if you have had certain experiences in your day-to-day life?  If yes, please tell me how often each of these might have occurred - frequently, sometimes, rarely, or never.   (CHECK ONE ANSWER FOR EACH QUESTION)  You are treated with less courtesy or respect than other people. | 1) Every day  2) Every week  3) A few times per year  4) A few times in my life  5) Never 99) DON'T KNOW / NOT SURE | R   U   M |
| **10** | You are treated with less courtesy or respect by your husband. | 1) Every day  2) Every week  3) A few times per year  4) A few times in my life  5) Never 99) DON'T KNOW / NOT SURE | R   U   M |
| **11** | People act as if they think you are not smart. | 1) Every day  2) Every week  3) A few times per year  4) A few times in my life  5) Never 99) DON'T KNOW / NOT SURE | R   U   M |
| **12** | People act as if they think you are dishonest. | 1) Every day  2) Every week  3) A few times per year  4) A few times in my life  5) Never 99) DON'T KNOW / NOT SURE | R   U   M |

| **13** | You are threatened or harassed. | | 1) Every day  2) Every week  3) A few times per year  4) A few times in my life  5) Never 99) DON'T KNOW / NOT SURE | R   U   M | |  |
| --- | --- | --- | --- | --- | --- | --- |
| **14** | Cancer testing or treatment that is unpleasant is worth getting if it would help me to live longer. | 1) Strongly Disagree 2) Disagree 3) Undecided 4) Agree 5) Strongly Agree | | | R   U   M | |
| **15** | If I had cancer, I would want to know that I have it. | 1) Strongly Disagree 2) Disagree 3) Undecided 4) Agree 5) Strongly Agree | | | R   U   M | |
| **16** | If I had cancer, I would want my family to know that I have it. | 1) Strongly Disagree 2) Disagree 3) Undecided 4) Agree 5) Strongly Agree | | | R   U   M | |
| **17** | If someone else in my family had cancer, I would want to know that they have it. | 1) Strongly Disagree 2) Disagree 3) Undecided 4) Agree 5) Strongly Agree | | | R   U   M | |
| **18** | Getting a serious disease like cancer is fate, there is nothing I can do to change fate. | 1) Strongly Disagree 2) Disagree 3) Undecided 4) Agree 5) Strongly Agree | | | R   U   M | |
| **19** | I would not feel comfortable around someone with cancer. | 1) Strongly Disagree 2) Disagree 3) Undecided 4) Agree 5) Strongly Agree | | | R   U   M | |
| **20** | Once you’ve had cancer you’re never ‘normal’ again. | 1) Strongly Disagree 2) Disagree 3) Undecided 4) Agree 5) Strongly Agree | | | R   U   M | |
| **21** | I would not sit or stand close to someone with cancer. | 1) Strongly Disagree 2) Disagree 3) Undecided 4) Agree 5) Strongly Agree | | | R   U   M | |
| **22** | The health care needs of people with cancer should not be prioritized. | 1) Strongly Disagree 2) Disagree 3) Undecided 4) Agree 5) Strongly Agree | | | R   U   M | |
| **23** | If a person has cancer it’s probably their fault. | 1) Strongly Disagree 2) Disagree 3) Undecided 4) Agree 5) Strongly Agree | | | R   U   M | |
| **24** | I would feel sorry for someone with cancer. | 1) Strongly Disagree 2) Disagree 3) Undecided 4) Agree 5) Strongly Agree | | | R   U   M | |
| **25** | Cancer is more frightening than most other diseases. | 1) Strongly Disagree 2) Disagree 3) Undecided 4) Agree 5) Strongly Agree | | | R   U   M | |
| **26** | Other women often state that they are worried about getting cancer. | 1) Strongly Disagree 2) Disagree 3) Undecided 4) Agree 5) Strongly Agree | | | R   U   M | |
| **27** | Overall, other women that I know recommend the cervical cancer test. | 1) Strongly Disagree 2) Disagree 3) Undecided 4) Agree 5) Strongly Agree | | | R   U   M | |
| **28** | I would recommend that other women get routine testing for cervical cancer. | 1) Strongly Disagree 2) Disagree 3) Undecided 4) Agree 5) Strongly Agree | | | R   U   M | |
| **29** | A woman’s most important role is to take care of her home and cook for her family. | 1) Strongly Disagree 2) Disagree 3) Undecided 4) Agree 5) Strongly Agree | | | R   U   M | |
| **30** | A man should have the final word about decisions in his home. | 1) Strongly Disagree 2) Disagree 3) Undecided 4) Agree 5) Strongly Agree | | | R   U   M | |
| **31** | Who in your family usually has the final say regarding the health of women at home? | 1) Yourself,  2) Husband/Partner,  3) Yourself/ husband /partner jointly,  4) Someone else,  5) You and someone else jointly  88) Other 99) DON'T KNOW / NOT SURE | | | R   U   M | |
| **32** | If "OTHER,"  Please specify. |  | | | R   U   M | |
| **33** | Who's opinion would most influence your decision to get or not to get a cervical cancer test? |  | | | R   U   M | |
