# Supplementary material for: Role of gender in perspectives of discrimination, stigma, and attitudes relative to cervical cancer in rural Sénégal
Source: PLoS One. 2020 Apr 28;15(4):e0232291. doi: 10.1371/journal.pone.0232291 (PMC7188246; doi:10.1371/journal.pone.0232291)
Supplement: S2 Questionnaire — (DOCX) [file pone.0232291.s005.docx]

***Des instructions destinées à l’enquêteur***

Laisser la case vide – Seulement réservé à un usage administratif

**No. du Participant: ______________**

**DCE#: DCI K011601.__ __ __**

**Data Entry Completion Date: _____**

**_______________**

- Veuillez clairement encercler le nombre qui se trouve à côté de la réponse donnée par la personne.
- Sauf indication contraire, veuillez encercler seulement une réponse pour chaque question.
- Sauf indication contraire (les instructions particulières concernant les questions sont en lettres MAJUSCULES), lisez la question et les réponses à la personne interrogée
- Les mots en lettres MAJUSCULES ne sont PAS lus, alors que les mots en lettres MINUSCULES sont lus à la personne interrogée.

**Refus de répondre, Mal à l’aise avec une Question, Incompréhension**

- Si la personne interrogée refuse de répondre à une question, veuillez encercler un (R)* à droite de l’espace réservé à la réponse
- Si la personne interrogée semble être mal à l’aise avec la question, veuillez encercler un (MA) * à droite de l’espace réservé à la réponse
- Si l’interlocuteur semble mal comprendre interprète mal la question, veuillez encercler un (I)* à droite de l’espace réservé à la réponse.
- Vous pouvez encercler plusieurs lettres pour toute question formulée.

**VEUILLEZ REMPLIR LES INFORMATION SUIVANTES POUR TOUTES LES ENTREVUES:**

| Admin 02 | ENQUETEUR |  |
| --- | --- | --- |
| Admin 03 | DATE | _____/ _____ / 20___ ( JJ / MM / YYYY ) |
| Admin 04 | HEURE DEBUTÉE |  |
| Admin 05 | HEURE TERMINEE |  |
| Admin 06 | No. de GROUPE | 1. SALEMATA DISTRICT 2. SARAYA DISTRICT 3. KEDOUGOU DISTRICT |
| Admin 07 | No.de COMMUNAUTE | SALEMATA DISTRICT 11 - Salemata 12 - Dar Salaam 13 - Dakately  SARAYA DISTRICT 21 - Saraya 22 - Nafadji 23 - Khossanto KEDOUGOU DISTRICT 31 - Kedougou - Dalaba  32 - Bandifassi 33 - Dindefello |
| Admin 08 | No.de MENAGE OU MEMBRE DE GROUPE DE SOUTIENNE |  |

| **1** | GENRE | | 0) FEMININ |  | |  |
| --- | --- | --- | --- | --- | --- | --- |
| **2** | Quel âge avez-vous? | |  |  | |  |
| **3** | Quelles langues préférez-vous parler? Préférez-vous …   (Donnez-moi seulement les langue(s) que vous préférez, sans tenir compte de la compétence)   (ENCERCLEZ TOUT CE QUI S’APPLIQUE) | | PLUSIEURS REPONSES POSSIBLES  1) Malinké  2) Pulaar  3) Basari  4) Bedick  5) Sérère  6) Wolof  7) Français  88) Autre  99) NE SAIS PAS / PAS CERTAIN | R   MA  I | |  |
| **4** | SI “AUTRES”  Veuillez préciser. | |  | R   MA  I | |  |
| **5** | Est-ce qu’un agent de santé vous a une fois fait subir un examen de dépistage de cancer du col de l’utérus? | | 0) Non, je n’ai jamais été dépistée  1) Oui, une fois seulement  2) Oui, plus d’une fois  99) NE SAIS PAS / PAS CERTAIN | R   MA  I | |  |
| **6** | Quel est le plus haut niveau d'éducation que vous avez complété? | | 0) Néant  1) Ecole Coranique  2) Education Primaire  3) Ecole Secondaire, jusqu’à deux ans  4) Ecole Secondaire, plus de deux ans  5) Un peu d’éducation universitaire  99) NE SAIS PAS / PAS CERTAIN | R   MA  I | |  |
| **7** | Quelle est votre situation matrimoniale? | | 1) Célibataire  2) Mariée (ménage monogame)  3) Mariée (ménage polygame)  4) Vivant comme marié / en cohabitation  5) Divorcée ou Séparée  6) Veuve  99) NE SAIS PAS / PAS CERTAIN | R   MA  I | |  |
| **8** | En qui avez-vous le plus confiance dans la prise de décisions concernant vos soins médicaux? | |  | R   MA  I | |  |
| **9** | Dans les questions suivantes, je vais vous demander si vous avez eu certaines expériences dans votre vie quotidienne? Si oui, veuillez me dire combien de fois chacun de ces cas peut se produire - fréquemment, parfois, rarement ou jamais.   (COCHEZ UNE RÉPONSE POUR CHAQUE QUESTION)   Vous êtes traité avec moins de courtoisie ou de respect que d'autres personnes. | | 1) Chaque jour  2) Chaque semaine  3) Quelquefois par année  4) Quelquefois dans ma vie  5) Jamais  99) NE SAIS PAS / PAS CERTAIN | R   MA  I | |  |
| **10** | Vous êtes traité avec moins de courtoisie ou de respect par votre époux. | | 1) Chaque jour  2) Chaque semaine  3) Quelquefois par année  4) Quelquefois dans ma vie  5) Jamais  99) NE SAIS PAS / PAS CERTAIN | R   MA  I | |  |
| **11** | Les gens agissent comme s’ils pensent que vous n'êtes pas intelligent. | | 1) Chaque jour  2) Chaque semaine  3) Quelquefois par année  4) Quelquefois dans ma vie  5) Jamais  99) NE SAIS PAS / PAS CERTAIN | R   MA  I | |  |
| **12** | Les gens agissent comme si ils pensent que vous êtes malhonnête. | | 1) Chaque jour  2) Chaque semaine  3) Quelquefois par année  4) Quelquefois dans ma vie  5) Jamais  99) NE SAIS PAS / PAS CERTAIN | R   MA  I | |  |
| **13** | Vous êtes menacé ou harcelé. | | 1) Chaque jour  2) Chaque semaine  3) Quelquefois par année  4) Quelquefois dans ma vie  5) Jamais  99) NE SAIS PAS / PAS CERTAIN | R   MA  I | |  |
| **14** | Subir un examen ou un traitement désagréable du cancer en vaut la peine si cela m'aiderait à vivre plus longtemps. | 1) Entièrement en désaccord  2) En désaccord  3) Indécis  4) D’accord  5) Entièrement d’accord | | | R   MA  I | |
| **15** | Si j'avais le cancer, je voudrais savoir que je l'ai. | 1) Entièrement en désaccord  2) En désaccord  3) Indécis  4) D’accord  5) Entièrement d’accord | | | R   MA  I | |
| **16** | Si j'avais le cancer, je voudrais que ma famille le sache. | 1) Entièrement en désaccord  2) En désaccord  3) Indécis  4) D’accord  5) Entièrement d’accord | | | R   MA  I | |
| **17** | Si quelqu'un d'autre dans ma famille avait le cancer, j’aurais voulu le savoir. | 1) Entièrement en désaccord  2) En désaccord  3) Indécis  4) D’accord  5) Entièrement d’accord | | | R   MA  I | |
| **18** | Avoir une maladie grave comme le cancer relève du destin, il n'y a rien que je puisse faire pour changer le destin. | 1) Entièrement en désaccord  2) En désaccord  3) Indécis  4) D’accord  5) Entièrement d’accord | | | R   MA  I | |
| **19** | Je ne me sentirais pas confortable aux côtés de quelqu'un qui a le cancer. | 1) Entièrement en désaccord  2) En désaccord  3) Indécis  4) D’accord  5) Entièrement d’accord | | | R   MA  I | |
| **20** | Une fois vous ayez eu le cancer, vous ne redevenez jamais 'normal'. | 1) Entièrement en désaccord  2) En désaccord  3) Indécis  4) D’accord  5) Entièrement d’accord | | | R   MA  I | |
| **21** | ne m'assiérais ou ne resterais pas debout auprès de quelqu'un qui a le cancer. | 1) Entièrement en désaccord  2) En désaccord  3) Indécis  4) D’accord  5) Entièrement d’accord | | | R   MA  I | |
| **22** | Les besoins en soins médicaux des personnes vivant avec le cancer ne devraient pas être privilégiés. | 1) Entièrement en désaccord  2) En désaccord  3) Indécis  4) D’accord  5) Entièrement d’accord | | | R   MA  I | |
| **23** | Si une personne a le cancer, c'est probablement de sa faute. | 1) Entièrement en désaccord  2) En désaccord  3) Indécis  4) D’accord  5) Entièrement d’accord | | | R   MA  I | |
| **24** | Je me sentirais désolé pour quelqu'un qui a le cancer. | 1) Entièrement en désaccord  2) En désaccord  3) Indécis  4) D’accord  5) Entièrement d’accord | | | R   MA  I | |
| **25** | Le cancer est plus effrayant que la plupart des autres maladies. | 1) Entièrement en désaccord  2) En désaccord  3) Indécis  4) D’accord  5) Entièrement d’accord | | | R   MA  I | |
| **26** | D’autres femmes affirment souvent qu'elles ont peur d’attraper le cancer. | 1) Entièrement en désaccord  2) En désaccord  3) Indécis  4) D’accord  5) Entièrement d’accord | | | R   MA  I | |
| **27** | En général, d’autres femmes que je connais recommandent l’examen de dépistage de cancer du col de l’utérus | 1) Entièrement en désaccord  2) En désaccord  3) Indécis  4) D’accord  5) Entièrement d’accord | | | R   MA  I | |
| **28** | Je recommanderais à d’autres femmes de subir un examen d’usage de dépistage de cancer du col de l’utérus. | 1) Entièrement en désaccord  2) En désaccord  3) Indécis  4) D’accord  5) Entièrement d’accord | | | R   MA  I | |
| **29** | Le rôle le plus important d'une femme est de prendre soin de sa maison et de cuisiner pour sa famille. | 1) Entièrement en désaccord  2) En désaccord  3) Indécis  4) D’accord  5) Entièrement d’accord | | | R   MA  I | |
| **30** | Un homme devrait avoir le dernier mot sur les décisions dans sa maison. | 1) Entièrement en désaccord  2) En désaccord  3) Indécis  4) D’accord  5) Entièrement d’accord | | | R   MA  I | |
| **31** | Qui dans votre famille a généralement le dernier mot concernant la santé des femmes à la maison? | 1) Vous-même,  2) Mari / partenaire,  3) vous-même / mari / partenaire conjointement,  4) Quelqu'un d'autre,  5) Vous et quelqu'un d'autre conjointement  88) Autre  99) NE SAIT PAS / PAS SÛR | | | R   MA  I | |
| **32** | Si “AUTRES” Veuillez préciser. |  | | | R  MA I | |
| **33** | L'opinion de quelle personne influencerait le plus votre décision de subir ou de ne pas subir un examen de dépistage de cancer du col de l’utérus ? |  | | | R   MA  I | |
