# Supplementary material for: Role of gender in perspectives of discrimination, stigma, and attitudes relative to cervical cancer in rural Sénégal
Source: PLoS One. 2020 Apr 28;15(4):e0232291. doi: 10.1371/journal.pone.0232291 (PMC7188246; doi:10.1371/journal.pone.0232291)
Supplement: S1 Table — (DOC) [file pone.0232291.s006.doc]

|  | Not Screened (N=85) | Screened (N=14) | Screened x2 (N=2) | Total (N=101) | p value |
| --- | --- | --- | --- | --- | --- |
| **Other women that I know recommend the cervical cancer test** |  |  |  |  | 0.117 |
| 1) Strongly Disagree | 1 (1.2%) | 1 (7.1%) | 0 (0.0%) | 2 (2.0%) |  |
| 2) Disagree | 2 (2.4%) | 2 (14.3%) | 0 (0.0%) | 4 (4.0%) |  |
| 3) Undecided | 28 (32.9%) | 2 (14.3%) | 0 (0.0%) | 30 (29.7%) |  |
| 4) Agree | 36 (42.4%) | 5 (35.7%) | 2 (100.0%) | 43 (42.6%) |  |
| 5) Strongly Agree | 18 (21.2%) | 4 (28.6%) | 0 (0.0%) | 22 (21.8%) |  |
| **I would recommend that women get routine testing for cervical cancer** |  |  |  |  | 0.007 |
| 1) Strongly Disagree | 2 (2.4%) | 1 (7.7%) | 0 (0.0%) | 3 (3.0%) |  |
| 2) Disagree | 3 (3.6%) | 0 (0.0%) | 0 (0.0%) | 3 (3.0%) |  |
| 3) Undecided | 11 (13.1%) | 0 (0.0%) | 0 (0.0%) | 11 (11.1%) |  |
| 4) Agree | 31 (36.9%) | 0 (0.0%) | 2 (100.0%) | 33 (33.3%) |  |
| 5) Strongly Agree | 37 (44.0%) | 12 (92.3%) | 0 (0.0%) | 49 (49.5%) |  |
| **A womans most important role is to take care of her home and cook for her family** |  |  |  |  | 0.257 |
| 1) Strongly Disagree | 5 (6.0%) | 1 (7.1%) | 0 (0.0%) | 6 (6.0%) |  |
| 2) Disagree | 21 (25.0%) | 3 (21.4%) | 2 (100.0%) | 26 (26.0%) |  |
| 3) Undecided | 1 (1.2%) | 1 (7.1%) | 0 (0.0%) | 2 (2.0%) |  |
| 4) Agree | 7 (8.3%) | 0 (0.0%) | 0 (0.0%) | 7 (7.0%) |  |
| 5) Strongly Agree | 50 (59.5%) | 9 (64.3%) | 0 (0.0%) | 59 (59.0%) |  |
| **A man should have the final word about decisions in his home** |  |  |  |  | 0.451 |
| 1) Strongly Disagree | 6 (7.1%) | 1 (7.1%) | 0 (0.0%) | 7 (6.9%) |  |
| 2) Disagree | 20 (23.5%) | 2 (14.3%) | 2 (100.0%) | 24 (23.8%) |  |
| 3) Undecided | 1 (1.2%) | 0 (0.0%) | 0 (0.0%) | 1 (1.0%) |  |
| 4) Agree | 12 (14.1%) | 3 (21.4%) | 0 (0.0%) | 15 (14.9%) |  |
| 5) Strongly Agree | 46 (54.1%) | 8 (57.1%) | 0 (0.0%) | 54 (53.5%) |  |
| **I would not feel comfortable around someone with cancer.** |  |  |  |  | < 0.001 |
| 1) Strongly Disagree | 4 (4.7%) | 6 (46.2%) | 1 (50.0%) | 11 (11.0%) |  |
| 2) Disagree | 41 (48.2%) | 2 (15.4%) | 1 (50.0%) | 44 (44.0%) |  |
| 3) Undecided | 0 (0.0%) | 1 (7.7%) | 0 (0.0%) | 1 (1.0%) |  |
| 4) Agree | 19 (22.4%) | 0 (0.0%) | 0 (0.0%) | 19 (19.0%) |  |
| 5) Strongly Agree | 21 (24.7%) | 4 (30.8%) | 0 (0.0%) | 25 (25.0%) |  |
| **Once youve had cancer youre never normal again.** |  |  |  |  | 0.266 |
| 1) Strongly Disagree | 1 (1.2%) | 1 (7.7%) | 0 (0.0%) | 2 (2.0%) |  |
| 2) Disagree | 7 (8.2%) | 1 (7.7%) | 0 (0.0%) | 8 (8.0%) |  |
| 3) Undecided | 2 (2.4%) | 0 (0.0%) | 0 (0.0%) | 2 (2.0%) |  |
| 4) Agree | 29 (34.1%) | 1 (7.7%) | 1 (50.0%) | 31 (31.0%) |  |
| 5) Strongly Agree | 46 (54.1%) | 10 (76.9%) | 1 (50.0%) | 57 (57.0%) |  |
| **The health care needs of people with cancer should not be prioritized.** |  |  |  |  | 0.202 |
| 1) Strongly Disagree | 6 (7.1%) | 3 (23.1%) | 0 (0.0%) | 9 (9.1%) |  |
| 2) Disagree | 20 (23.8%) | 3 (23.1%) | 1 (50.0%) | 24 (24.2%) |  |
| 3) Undecided | 3 (3.6%) | 0 (0.0%) | 0 (0.0%) | 3 (3.0%) |  |
| 4) Agree | 29 (34.5%) | 1 (7.7%) | 1 (50.0%) | 31 (31.3%) |  |
| 5) Strongly Agree | 26 (31.0%) | 6 (46.2%) | 0 (0.0%) | 32 (32.3%) |  |
| **If a person has cancer its probably their fault.** |  |  |  |  | 0.001 |
| 1) Strongly Disagree | 8 (9.4%) | 3 (23.1%) | 0 (0.0%) | 11 (11.0%) |  |
| 2) Disagree | 38 (44.7%) | 1 (7.7%) | 1 (50.0%) | 40 (40.0%) |  |
| 3) Undecided | 4 (4.7%) | 1 (7.7%) | 1 (50.0%) | 6 (6.0%) |  |
| 4) Agree | 16 (18.8%) | 0 (0.0%) | 0 (0.0%) | 16 (16.0%) |  |
| 5) Strongly Agree | 19 (22.4%) | 8 (61.5%) | 0 (0.0%) | 27 (27.0%) |  |
| **I would feel sorry for someone with cancer.** |  |  |  |  | 0.451 |
| 1) Strongly Disagree | 1 (1.2%) | 0 (0.0%) | 0 (0.0%) | 1 (1.0%) |  |
| 2) Disagree | 9 (10.6%) | 0 (0.0%) | 0 (0.0%) | 9 (9.0%) |  |
| 4) Agree | 41 (48.2%) | 5 (38.5%) | 2 (100.0%) | 48 (48.0%) |  |
| 5) Strongly Agree | 34 (40.0%) | 8 (61.5%) | 0 (0.0%) | 42 (42.0%) |  |
| **I feel that cancer is more frightening than most other diseases.** |  |  |  |  | 0.368 |
| 2) Disagree | 9 (10.7%) | 1 (7.7%) | 1 (50.0%) | 11 (11.1%) |  |
| 3) Undecided | 4 (4.8%) | 0 (0.0%) | 0 (0.0%) | 4 (4.0%) |  |
| 4) Agree | 30 (35.7%) | 3 (23.1%) | 1 (50.0%) | 34 (34.3%) |  |
| 5) Strongly Agree | 41 (48.8%) | 9 (69.2%) | 0 (0.0%) | 50 (50.5%) |  |
| **Other women often state that they are worried about getting cancer.** |  |  |  |  | 0.018 |
| 2) Disagree | 2 (2.4%) | 1 (7.7%) | 0 (0.0%) | 3 (3.0%) |  |
| 3) Undecided | 22 (25.9%) | 2 (15.4%) | 0 (0.0%) | 24 (24.0%) |  |
| 4) Agree | 23 (27.1%) | 0 (0.0%) | 2 (100.0%) | 25 (25.0%) |  |
| 5) Strongly Agree | 38 (44.7%) | 10 (76.9%) | 0 (0.0%) | 48 (48.0%) |  |
| **Cancer testing or treatment that is unpleasant is worth getting if it would help me to live longer** |  |  |  |  | 0.248 |
| 1) Strongly Disagree | 2 (2.4%) | 0 (0.0%) | 0 (0.0%) | 2 (2.0%) |  |
| 2) Disagree | 7 (8.2%) | 1 (7.7%) | 0 (0.0%) | 8 (8.0%) |  |
| 3) Undecided | 9 (10.6%) | 0 (0.0%) | 0 (0.0%) | 9 (9.0%) |  |
| 4) Agree | 39 (45.9%) | 3 (23.1%) | 2 (100.0%) | 44 (44.0%) |  |
| 5) Strongly Agree | 28 (32.9%) | 9 (69.2%) | 0 (0.0%) | 37 (37.0%) |  |
| **If I had cancer, I would want to know that I have it** |  |  |  |  | 0.038 |
| 1) Strongly Disagree | 1 (1.2%) | 0 (0.0%) | 0 (0.0%) | 1 (1.0%) |  |
| 2) Disagree | 5 (5.9%) | 0 (0.0%) | 0 (0.0%) | 5 (5.1%) |  |
| 4) Agree | 37 (43.5%) | 1 (8.3%) | 2 (100.0%) | 40 (40.4%) |  |
| 5) Strongly Agree | 42 (49.4%) | 11 (91.7%) | 0 (0.0%) | 53 (53.5%) |  |
| **If I had cancer, I would want my family to know that I have it.** |  |  |  |  | 0.089 |
| 1) Strongly Disagree | 7 (8.2%) | 3 (23.1%) | 0 (0.0%) | 10 (10.0%) |  |
| 2) Disagree | 15 (17.6%) | 3 (23.1%) | 0 (0.0%) | 18 (18.0%) |  |
| 3) Undecided | 3 (3.5%) | 0 (0.0%) | 0 (0.0%) | 3 (3.0%) |  |
| 4) Agree | 36 (42.4%) | 1 (7.7%) | 2 (100.0%) | 39 (39.0%) |  |
| 5) Strongly Agree | 24 (28.2%) | 6 (46.2%) | 0 (0.0%) | 30 (30.0%) |  |
| **If someone else in my family had cancer, I would want to know that they have it.** |  |  |  |  | 0.050 |
| 1) Strongly Disagree | 2 (2.4%) | 2 (15.4%) | 0 (0.0%) | 4 (4.0%) |  |
| 2) Disagree | 23 (27.4%) | 2 (15.4%) | 0 (0.0%) | 25 (25.3%) |  |
| 4) Agree | 34 (40.5%) | 2 (15.4%) | 2 (100.0%) | 38 (38.4%) |  |
| 5) Strongly Agree | 25 (29.8%) | 7 (53.8%) | 0 (0.0%) | 32 (32.3%) |  |
| **Getting a serious disease like cancer is fate, there is nothing I can do to change fate** |  |  |  |  | 0.013 |
| 1) Strongly Disagree | 16 (18.8%) | 8 (61.5%) | 0 (0.0%) | 24 (24.0%) |  |
| 2) Disagree | 23 (27.1%) | 3 (23.1%) | 1 (50.0%) | 27 (27.0%) |  |
| 3) Undecided | 6 (7.1%) | 0 (0.0%) | 0 (0.0%) | 6 (6.0%) |  |
| 4) Agree | 29 (34.1%) | 0 (0.0%) | 1 (50.0%) | 30 (30.0%) |  |
| 5) Strongly Agree | 11 (12.9%) | 2 (15.4%) | 0 (0.0%) | 13 (13.0%) |  |
| **DSz_HCSN** |  |  |  |  | 0.828 |
| 0 | 69 (83.1%) | 11 (78.6%) | 2 (100.0%) | 82 (82.8%) |  |
| 1 | 13 (15.7%) | 3 (21.4%) | 0 (0.0%) | 16 (16.2%) |  |
| 2 | 1 (1.2%) | 0 (0.0%) | 0 (0.0%) | 1 (1.0%) |  |
| **Feel treated with less courtesy or respect than others** |  |  |  |  | 0.761 |
| 1) Every day | 9 (10.8%) | 0 (0.0%) | 0 (0.0%) | 9 (9.2%) |  |
| 2) Every week | 2 (2.4%) | 0 (0.0%) | 0 (0.0%) | 2 (2.0%) |  |
| 3) A few times per year | 6 (7.2%) | 0 (0.0%) | 0 (0.0%) | 6 (6.1%) |  |
| 4) A few times in my life | 17 (20.5%) | 4 (30.8%) | 1 (50.0%) | 22 (22.4%) |  |
| 5) Never | 49 (59.0%) | 9 (69.2%) | 1 (50.0%) | 59 (60.2%) |  |
| **Feel treated with less courtesy or respect by their spouse** |  |  |  |  | 0.875 |
| 1) Every day | 11 (13.3%) | 3 (23.1%) | 0 (0.0%) | 14 (14.3%) |  |
| 2) Every week | 4 (4.8%) | 0 (0.0%) | 0 (0.0%) | 4 (4.1%) |  |
| 3) A few times per year | 14 (16.9%) | 1 (7.7%) | 0 (0.0%) | 15 (15.3%) |  |
| 4) A few times in my life | 15 (18.1%) | 2 (15.4%) | 1 (50.0%) | 18 (18.4%) |  |
| 5) Never | 39 (47.0%) | 7 (53.8%) | 1 (50.0%) | 47 (48.0%) |  |
| **Feel that others act as if they are not smart** |  |  |  |  | 0.595 |
| 1) Every day | 1 (1.2%) | 0 (0.0%) | 0 (0.0%) | 1 (1.0%) |  |
| 2) Every week | 1 (1.2%) | 0 (0.0%) | 0 (0.0%) | 1 (1.0%) |  |
| 3) A few times per year | 4 (4.7%) | 0 (0.0%) | 0 (0.0%) | 4 (4.0%) |  |
| 4) A few times in my life | 14 (16.5%) | 1 (7.1%) | 1 (50.0%) | 16 (15.8%) |  |
| 5) Never | 65 (76.5%) | 13 (92.9%) | 1 (50.0%) | 79 (78.2%) |  |
| **Feel perceived as being dishonest** |  |  |  |  | 0.360 |
| 3) A few times per year | 1 (1.2%) | 0 (0.0%) | 0 (0.0%) | 1 (1.0%) |  |
| 4) A few times in my life | 9 (10.7%) | 1 (7.1%) | 1 (50.0%) | 11 (11.0%) |  |
| 5) Never | 74 (88.1%) | 13 (92.9%) | 1 (50.0%) | 88 (88.0%) |  |
| **Feel threatened by others** |  |  |  |  | 0.379 |
| 1) Every day | 1 (1.2%) | 0 (0.0%) | 0 (0.0%) | 1 (1.0%) |  |
| 3) A few times per year | 2 (2.4%) | 1 (7.1%) | 0 (0.0%) | 3 (3.0%) |  |
| 4) A few times in my life | 14 (16.9%) | 1 (7.1%) | 1 (50.0%) | 16 (16.2%) |  |
| 5) Never | 66 (79.5%) | 12 (85.7%) | 1 (50.0%) | 79 (79.8%) |  |
| **You have the final say at home regarding your healthcare decisions** |  |  |  |  | 0.013 |
| No | 73 (86.9%) | 11 (78.6%) | 0 (0.0%) | 84 (84.0%) |  |
| Yes | 11 (13.1%) | 3 (21.4%) | 2 (100.0%) | 16 (16.0%) |  |
| **Your spouse has the final say at home regarding your healthcare decisions** |  |  |  |  | 0.057 |
| No | 21 (25.0%) | 5 (35.7%) | 2 (100.0%) | 28 (28.0%) |  |
| Yes | 63 (75.0%) | 9 (64.3%) | 0 (0.0%) | 72 (72.0%) |  |
| **Other family members are most likely to influence your decision to get screened for cervical cancer** |  |  |  |  | 0.472 |
| No | 76 (89.4%) | 14 (100.0%) | 2 (100.0%) | 92 (91.1%) |  |
| Yes | 9 (10.6%) | 0 (0.0%) | 0 (0.0%) | 9 (8.9%) |  |
| **The head of the household is most likely to influence your decision to get screened for cervical cancer** |  |  |  |  | 0.413 |
| No | 37 (43.5%) | 6 (42.9%) | 2 (100.0%) | 45 (44.6%) |  |
| Yes | 48 (56.5%) | 8 (57.1%) | 0 (0.0%) | 56 (55.4%) |  |
| **Your spouse is most likely to influence your decision to get screened for cervical cancer** |  |  |  |  | 0.291 |
| No | 81 (95.3%) | 12 (85.7%) | 2 (100.0%) | 95 (94.1%) |  |
| Yes | 4 (4.7%) | 2 (14.3%) | 0 (0.0%) | 6 (5.9%) |  |
| **The nurse is most likely to influence your decision to get screened for cervical cancer** |  |  |  |  | 1.000 |
| No | 84 (98.8%) | 14 (100.0%) | 2 (100.0%) | 100 (99.0%) |  |
| Yes | 1 (1.2%) | 0 (0.0%) | 0 (0.0%) | 1 (1.0%) |  |
| **No one other than you will influence your decision to get screened for cervical cancer** |  |  |  |  | 0.114 |
| No | 56 (65.9%) | 11 (78.6%) | 0 (0.0%) | 67 (66.3%) |  |
| Yes | 29 (34.1%) | 3 (21.4%) | 2 (100.0%) | 34 (33.7%) |  |
| **When making decisions about your healthcare, you most trust your spouse** |  |  |  |  | 0.363 |
| No | 43 (50.6%) | 9 (64.3%) | 2 (100.0%) | 54 (53.5%) |  |
| Yes | 42 (49.4%) | 5 (35.7%) | 0 (0.0%) | 47 (46.5%) |  |
| **When making decisions about your healthcare, you most trust your nurse** |  |  |  |  | 0.321 |
| No | 70 (82.4%) | 11 (78.6%) | 1 (50.0%) | 82 (81.2%) |  |
| Yes | 15 (17.6%) | 3 (21.4%) | 1 (50.0%) | 19 (18.8%) |  |
| **When making decisions about your healthcare, you most trust other family members** |  |  |  |  | 0.506 |
| No | 71 (83.5%) | 10 (71.4%) | 2 (100.0%) | 83 (82.2%) |  |
| Yes | 14 (16.5%) | 4 (28.6%) | 0 (0.0%) | 18 (17.8%) |  |
| **When making decisions about your healthcare, you most trust your physician** |  |  |  |  | 0.291 |
| No | 84 (98.8%) | 13 (92.9%) | 2 (100.0%) | 99 (98.0%) |  |
| Yes | 1 (1.2%) | 1 (7.1%) | 0 (0.0%) | 2 (2.0%) |  |
| **When making decisions about your healthcare, you most trust the community health worker** |  |  |  |  |  |
| No | 85 (100.0%) | 14 (100.0%) | 2 (100.0%) | 101 (100.0%) |  |
| **When making decisions about your healthcare, you most trust your children** |  |  |  |  | 0.117 |
| No | 75 (88.2%) | 14 (100.0%) | 1 (50.0%) | 90 (89.1%) |  |
| Yes | 10 (11.8%) | 0 (0.0%) | 1 (50.0%) | 11 (10.9%) |  |
| **When making decisions about your healthcare, you most trust yourself** |  |  |  |  | 1.000 |
| No | 78 (91.8%) | 13 (92.9%) | 2 (100.0%) | 93 (92.1%) |  |
| Yes | 7 (8.2%) | 1 (7.1%) | 0 (0.0%) | 8 (7.9%) |  |
