# Supplementary material for: Role of gender in perspectives of discrimination, stigma, and attitudes relative to cervical cancer in rural Sénégal
Source: PLoS One. 2020 Apr 28;15(4):e0232291. doi: 10.1371/journal.pone.0232291 (PMC7188246; doi:10.1371/journal.pone.0232291)
Supplement: S2 Table — (DOC) [file pone.0232291.s007.doc]

|  | Female  Low Education  (N=61) | Male  Low  Education (N=26) | Female Higher  Education (N=40) | Male  Higher  Education (N=30) | Total (N=157) | p value |
| --- | --- | --- | --- | --- | --- | --- |
| **A woman’s most important role is to take care of her home and cook for her family** |  |  |  |  |  | < 0.001 |
| Strongly Disagree | 4 (6.7%) | 1 (3.8%) | 2 (5.0%) | 0 (0.0%) | 7 (4.5%) |  |
| Disagree | 7 (11.7%) | 1 (3.8%) | 19 (47.5%) | 5 (16.7%) | 32 (20.5%) |  |
| Undecided | 2 (3.3%) | 2 (7.7%) | 0 (0.0%) | 0 (0.0%) | 4 (2.6%) |  |
| Agree | 6 (10.0%) | 14 (53.8%) | 1 (2.5%) | 10 (33.3%) | 31 (19.9%) |  |
| Strongly Agree | 41 (68.3%) | 8 (30.8%) | 18 (45.0%) | 15 (50.0%) | 82 (52.6%) |  |
| **A man should have the final word about decisions in his home** |  |  |  |  |  | < 0.001 |
| Strongly Disagree | 7 (11.5%) | 1 (3.8%) | 0 (0.0%) | 0 (0.0%) | 8 (5.1%) |  |
| Disagree | 9 (14.8%) | 0 (0.0%) | 15 (37.5%) | 3 (10.3%) | 27 (17.3%) |  |
| Undecided | 1 (1.6%) | 0 (0.0%) | 0 (0.0%) | 0 (0.0%) | 1 (0.6%) |  |
| Agree | 9 (14.8%) | 11 (42.3%) | 6 (15.0%) | 1 (3.4%) | 27 (17.3%) |  |
| Strongly Agree | 35 (57.4%) | 14 (53.8%) | 19 (47.5%) | 25 (86.2%) | 93 (59.6%) |  |
