# Supplementary material for: Role of gender in perspectives of discrimination, stigma, and attitudes relative to cervical cancer in rural Sénégal
Source: PLoS One. 2020 Apr 28;15(4):e0232291. doi: 10.1371/journal.pone.0232291 (PMC7188246; doi:10.1371/journal.pone.0232291)
Supplement: S3 Table — (DOC) [file pone.0232291.s008.doc]

|  | Female  Low Education  (N=61) | Male  Low  Education (N=26) | Female Higher  Education (N=40) | Male  Higher  Education (N=30) | Total (N=157) | p value |
| --- | --- | --- | --- | --- | --- | --- |
| **Feel treated with less courtesy or respect than others** |  |  |  |  |  | 0.008 |
| Every day | 6 (10.2%) | 0 (0.0%) | 3 (7.7%) | 0 (0.0%) | 9 (6.0%) |  |
| Every week | 1 (1.7%) | 0 (0.0%) | 1 (2.6%) | 0 (0.0%) | 2 (1.3%) |  |
| A few times per year | 6 (10.2%) | 0 (0.0%) | 0 (0.0%) | 3 (10.3%) | 9 (6.0%) |  |
| A few times in my life | 7 (11.9%) | 3 (12.5%) | 15 (38.5%) | 7 (24.1%) | 32 (21.2%) |  |
| Never | 39 (66.1%) | 21 (87.5%) | 20 (51.3%) | 19 (65.5%) | 99 (65.6%) |  |
| **Feel treated with less courtesy or respect by their spouse** |  |  |  |  |  | 0.010 |
| Every day | 10 (16.9%) | 0 (0.0%) | 4 (10.3%) | 0 (0.0%) | 14 (9.4%) |  |
| Every week | 4 (6.8%) | 0 (0.0%) | 0 (0.0%) | 0 (0.0%) | 4 (2.7%) |  |
| A few times per year | 9 (15.3%) | 1 (4.5%) | 6 (15.4%) | 1 (3.4%) | 17 (11.4%) |  |
| A few times in my life | 7 (11.9%) | 5 (22.7%) | 11 (28.2%) | 4 (13.8%) | 27 (18.1%) |  |
| Never | 29 (49.2%) | 16 (72.7%) | 18 (46.2%) | 24 (82.8%) | 87 (58.4%) |  |
| **Feel that others act as if they are not smart** |  |  |  |  |  | < 0.001 |
| Every day | 1 (1.6%) | 0 (0.0%) | 0 (0.0%) | 0 (0.0%) | 1 (0.7%) |  |
| Every week | 0 (0.0%) | 0 (0.0%) | 1 (2.5%) | 0 (0.0%) | 1 (0.7%) |  |
| A few times per year | 3 (4.9%) | 3 (12.5%) | 1 (2.5%) | 1 (3.6%) | 8 (5.2%) |  |
| A few times in my life | 3 (4.9%) | 6 (25.0%) | 13 (32.5%) | 11 (39.3%) | 33 (21.6%) |  |
| Never | 54 (88.5%) | 15 (62.5%) | 25 (62.5%) | 16 (57.1%) | 110 (71.9%) |  |
| **Feel perceived as being dishonest** |  |  |  |  |  | 0.008 |
| Every day | 0 (0.0%) | 0 (0.0%) | 0 (0.0%) | 0 (0.0%) | 0 (0.0%) |  |
| Every week | 0 (0.0%) | 0 (0.0%) | 0 (0.0%) | 0 (0.0%) | 0 (0.0%) |  |
| A few times per year | 1 (1.7%) | 0 (0.0%) | 0 (0.0%) | 1 (3.7%) | 2 (1.4%) |  |
| A few times in my life | 2 (3.3%) | 5 (25.0%) | 9 (22.5%) | 5 (18.5%) | 21 (14.3%) |  |
| Never | 57 (95.0%) | 15 (75.0%) | 31 (77.5%) | 21 (77.8%) | 124 (84.4%) |  |
| **Feel threatened by others** |  |  |  |  |  | 0.042 |
| Every day | 1 (1.7%) | 0 (0.0%) | 0 (0.0%) | 0 (0.0%) | 1 (0.7%) |  |
| Every week | 0 (0.0%) | 0 (0.0%) | 0 (0.0%) | 0 (0.0%) | 0 (0.0%) |  |
| A few times per year | 3 (5.1%) | 1 (4.8%) | 0 (0.0%) | 2 (7.4%) | 6 (4.1%) |  |
| A few times in my life | 6 (10.2%) | 4 (19.0%) | 10 (25.0%) | 0 (0.0%) | 20 (13.6%) |  |
| Never | 49 (83.1%) | 16 (76.2%) | 30 (75.0%) | 25 (92.6%) | 120 (81.6%) |  |
