# Supplementary material for: Role of gender in perspectives of discrimination, stigma, and attitudes relative to cervical cancer in rural Sénégal
Source: PLoS One. 2020 Apr 28;15(4):e0232291. doi: 10.1371/journal.pone.0232291 (PMC7188246; doi:10.1371/journal.pone.0232291)
Supplement: S4 Table — (DOC) [file pone.0232291.s009.doc]

|  | Female  Low Education  (N=61) | Male  Low  Education (N=26) | Female Higher  Education (N=40) | Male  Higher  Education (N=30) | Total (N=157) | p value |
| --- | --- | --- | --- | --- | --- | --- |
| **I would not feel comfortable around someone with cancer.** |  |  |  |  |  | < 0.001 |
| Strongly Disagree | 7 (11.5%) | 2 (7.7%) | 4 (10.3%) | 9 (31.0%) | 22 (14.2%) |  |
| Disagree | 21 (34.4%) | 20 (76.9%) | 23 (59.0%) | 11 (37.9%) | 75 (48.4%) |  |
| Undecided | 0 (0.0%) | 2 (7.7%) | 1 (2.6%) | 1 (3.4%) | 4 (2.6%) |  |
| Agree | 13 (21.3%) | 2 (7.7%) | 6 (15.4%) | 7 (24.1%) | 28 (18.1%) |  |
| Strongly Agree | 20 (32.8%) | 0 (0.0%) | 5 (12.8%) | 1 (3.4%) | 26 (16.8%) |  |
| **Once you’ve had cancer you’re never normal again.** |  |  |  |  |  | < 0.001 |
| Strongly Disagree | 1 (1.6%) | 1 (3.8%) | 1 (2.6%) | 6 (20.7%) | 9 (5.8%) |  |
| Disagree | 3 (4.9%) | 6 (23.1%) | 5 (12.8%) | 5 (17.2%) | 19 (12.3%) |  |
| Undecided | 1 (1.6%) | 6 (23.1%) | 1 (2.6%) | 3 (10.3%) | 11 (7.1%) |  |
| Agree | 18 (29.5%) | 10 (38.5%) | 13 (33.3%) | 13 (44.8%) | 54 (34.8%) |  |
| Strongly Agree | 38 (62.3%) | 3 (11.5%) | 19 (48.7%) | 2 (6.9%) | 62 (40.0%) |  |
| **The health care needs of people with cancer should not be prioritized.** |  |  |  |  |  | < 0.001 |
| Strongly Disagree | 7 (11.5%) | 5 (19.2%) | 2 (5.3%) | 13 (44.8%) | 27 (17.5%) |  |
| Disagree | 14 (23.0%) | 18 (69.2%) | 10 (26.3%) | 12 (41.4%) | 54 (35.1%) |  |
| Undecided | 3 (4.9%) | 1 (3.8%) | 0 (0.0%) | 1 (3.4%) | 5 (3.2%) |  |
| Agree | 17 (27.9%) | 2 (7.7%) | 14 (36.8%) | 0 (0.0%) | 33 (21.4%) |  |
| Strongly Agree | 20 (32.8%) | 0 (0.0%) | 12 (31.6%) | 3 (10.3%) | 35 (22.7%) |  |
| **If a person has cancer its probably their fault.** |  |  |  |  |  | < 0.001 |
| Strongly Disagree | 8 (13.1%) | 4 (15.4%) | 3 (7.7%) | 7 (24.1%) | 22 (14.2%) |  |
| Disagree | 17 (27.9%) | 13 (50.0%) | 23 (59.0%) | 10 (34.5%) | 63 (40.6%) |  |
| Undecided | 3 (4.9%) | 8 (30.8%) | 3 (7.7%) | 5 (17.2%) | 19 (12.3%) |  |
| Agree | 13 (21.3%) | 1 (3.8%) | 3 (7.7%) | 6 (20.7%) | 23 (14.8%) |  |
| Strongly Agree | 20 (32.8%) | 0 (0.0%) | 7 (17.9%) | 1 (3.4%) | 28 (18.1%) |  |
| **I would feel sorry for someone with cancer.** |  |  |  |  |  | 0.096 |
| Strongly Disagree | 0 (0.0%) | 2 (7.7%) | 1 (2.6%) | 2 (6.9%) | 5 (3.2%) |  |
| Disagree | 9 (14.8%) | 1 (3.8%) | 0 (0.0%) | 2 (6.9%) | 12 (7.7%) |  |
| Undecided | 0 (0.0%) | 0 (0.0%) | 0 (0.0%) | 0 (0.0%) | 0 (0.0%) |  |
| Agree | 28 (45.9%) | 12 (46.2%) | 20 (51.3%) | 15 (51.7%) | 75 (48.4%) |  |
| Strongly Agree | 24 (39.3%) | 11 (42.3%) | 18 (46.2%) | 10 (34.5%) | 63 (40.6%) |  |
| **I feel that cancer is more frightening than most other diseases.** |  |  |  |  |  | < 0.001 |
| Strongly Disagree | 0 (0.0%) | 1 (3.8%) | 0 (0.0%) | 5 (17.9%) | 6 (3.9%) |  |
| Disagree | 5 (8.2%) | 4 (15.4%) | 6 (15.8%) | 11 (39.3%) | 26 (17.0%) |  |
| Undecided | 2 (3.3%) | 3 (11.5%) | 2 (5.3%) | 2 (7.1%) | 9 (5.9%) |  |
| Agree | 24 (39.3%) | 11 (42.3%) | 10 (26.3%) | 8 (28.6%) | 53 (34.6%) |  |
| Strongly Agree | 30 (49.2%) | 7 (26.9%) | 20 (52.6%) | 2 (7.1%) | 59 (38.6%) |  |
| **Other women often state that they are worried about getting cancer.** |  |  |  |  |  | < 0.001 |
| Strongly Disagree | 0 (0.0%) | 1 (3.8%) | 0 (0.0%) | 0 (0.0%) | 1 (0.6%) |  |
| Disagree | 2 (3.3%) | 0 (0.0%) | 1 (2.6%) | 1 (3.4%) | 4 (2.6%) |  |
| Undecided | 11 (18.0%) | 15 (57.7%) | 13 (33.3%) | 14 (48.3%) | 53 (34.2%) |  |
| Agree | 12 (19.7%) | 8 (30.8%) | 13 (33.3%) | 12 (41.4%) | 45 (29.0%) |  |
| Strongly Agree | 36 (59.0%) | 2 (7.7%) | 12 (30.8%) | 2 (6.9%) | 52 (33.5%) |  |
