# Supplementary material for: Role of gender in perspectives of discrimination, stigma, and attitudes relative to cervical cancer in rural Sénégal
Source: PLoS One. 2020 Apr 28;15(4):e0232291. doi: 10.1371/journal.pone.0232291 (PMC7188246; doi:10.1371/journal.pone.0232291)
Supplement: S5 Table — (DOC) [file pone.0232291.s010.doc]

|  | Female  Low Education  (N=61) | Male  Low  Education (N=26) | Female Higher  Education (N=40) | Male  Higher  Education (N=30) | Total (N=157) | p value |
| --- | --- | --- | --- | --- | --- | --- |
| **Cancer testing or treatment that is unpleasant is worth getting if it would help me to live longer.** |  |  |  |  |  | 0.011 |
| Strongly Disagree | 2 (3.3%) | 1 (3.8%) | 0 (0.0%) | 0 (0.0%) | 3 (1.9%) |  |
| Disagree | 2 (3.3%) | 0 (0.0%) | 6 (15.4%) | 0 (0.0%) | 8 (5.2%) |  |
| Undecided | 4 (6.6%) | 2 (7.7%) | 5 (12.8%) | 4 (14.3%) | 15 (9.7%) |  |
| Agree | 25 (41.0%) | 19 (73.1%) | 19 (48.7%) | 15 (53.6%) | 78 (50.6%) |  |
| Strongly Agree | 28 (45.9%) | 4 (15.4%) | 9 (23.1%) | 9 (32.1%) | 50 (32.5%) |  |
| **If I had cancer, I would want to know that I have it.** |  |  |  |  |  | 0.024 |
| Strongly Disagree | 0 (0.0%) | 1 (3.8%) | 1 (2.6%) | 0 (0.0%) | 2 (1.3%) |  |
| Disagree | 2 (3.3%) | 0 (0.0%) | 3 (7.7%) | 0 (0.0%) | 5 (3.2%) |  |
| Undecided | 0 (0.0%) | 1 (3.8%) | 0 (0.0%) | 0 (0.0%) | 1 (0.6%) |  |
| Agree | 23 (38.3%) | 12 (46.2%) | 17 (43.6%) | 5 (17.2%) | 57 (37.0%) |  |
| Strongly Agree | 35 (58.3%) | 12 (46.2%) | 18 (46.2%) | 24 (82.8%) | 89 (57.8%) |  |
| **If I had cancer, I would want my family to know that I have it.** |  |  |  |  |  | 0.002 |
| Strongly Disagree | 9 (14.8%) | 1 (3.8%) | 1 (2.6%) | 0 (0.0%) | 11 (7.1%) |  |
| Disagree | 8 (13.1%) | 0 (0.0%) | 10 (25.6%) | 2 (6.9%) | 20 (12.9%) |  |
| Undecided | 2 (3.3%) | 1 (3.8%) | 1 (2.6%) | 2 (6.9%) | 6 (3.9%) |  |
| Agree | 24 (39.3%) | 11 (42.3%) | 15 (38.5%) | 5 (17.2%) | 55 (35.5%) |  |
| Strongly Agree | 18 (29.5%) | 13 (50.0%) | 12 (30.8%) | 20 (69.0%) | 63 (40.6%) |  |
| **If someone else in my family had cancer, I would want to know that they have it.** |  |  |  |  |  | < 0.001 |
| Strongly Disagree | 3 (5.0%) | 1 (3.8%) | 1 (2.6%) | 0 (0.0%) | 5 (3.2%) |  |
| Disagree | 15 (25.0%) | 0 (0.0%) | 10 (25.6%) | 0 (0.0%) | 25 (16.2%) |  |
| Undecided | 0 (0.0%) | 0 (0.0%) | 0 (0.0%) | 0 (0.0%) | 0 (0.0%) |  |
| Agree | 19 (31.7%) | 16 (61.5%) | 19 (48.7%) | 6 (20.7%) | 60 (39.0%) |  |
| Strongly Agree | 23 (38.3%) | 9 (34.6%) | 9 (23.1%) | 23 (79.3%) | 64 (41.6%) |  |
| **Getting a serious disease like cancer is fate, there is nothing I can do to change fate** |  |  |  |  |  | < 0.001 |
| Strongly Disagree | 21 (34.4%) | 2 (7.7%) | 3 (7.7%) | 9 (31.0%) | 35 (22.6%) |  |
| Disagree | 11 (18.0%) | 1 (3.8%) | 16 (41.0%) | 9 (31.0%) | 37 (23.9%) |  |
| Undecided | 3 (4.9%) | 8 (30.8%) | 3 (7.7%) | 1 (3.4%) | 15 (9.7%) |  |
| Agree | 21 (34.4%) | 14 (53.8%) | 9 (23.1%) | 6 (20.7%) | 50 (32.3%) |  |
| Strongly Agree | 5 (8.2%) | 1 (3.8%) | 8 (20.5%) | 4 (13.8%) | 18 (11.6%) |  |
