# Supplementary material for: Role of gender in perspectives of discrimination, stigma, and attitudes relative to cervical cancer in rural Sénégal
Source: PLoS One. 2020 Apr 28;15(4):e0232291. doi: 10.1371/journal.pone.0232291 (PMC7188246; doi:10.1371/journal.pone.0232291)
Supplement: S6 Table — (DOC) [file pone.0232291.s011.doc]

|  | Female  Low Education  (N=61) | Male  Low  Education (N=26) | Female Higher  Education (N=40) | Male  Higher  Education (N=30) | Total (N=157) | p value |
| --- | --- | --- | --- | --- | --- | --- |
| **Other women that I know recommend the cervical cancer test.** |  |  |  |  |  | 0.142 |
| Strongly Disagree | 1 (1.6%) | 1 (3.8%) | 1 (2.5%) | 0 (0.0%) | 3 (1.9%) |  |
| Disagree | 4 (6.6%) | 1 (3.8%) | 0 (0.0%) | 2 (6.9%) | 7 (4.5%) |  |
| Undecided | 13 (21.3%) | 8 (30.8%) | 17 (42.5%) | 12 (41.4%) | 50 (32.1%) |  |
| Agree | 26 (42.6%) | 9 (34.6%) | 17 (42.5%) | 13 (44.8%) | 65 (41.7%) |  |
| Strongly Agree | 17 (27.9%) | 7 (26.9%) | 5 (12.5%) | 2 (6.9%) | 31 (19.9%) |  |
| **I would recommend that women get routine testing for cervical cancer.** |  |  |  |  |  | 0.735 |
| Strongly Disagree | 2 (3.3%) | 1 (3.8%) | 1 (2.6%) | 0 (0.0%) | 4 (2.6%) |  |
| Disagree | 2 (3.3%) | 0 (0.0%) | 1 (2.6%) | 0 (0.0%) | 3 (1.9%) |  |
| Undecided | 5 (8.3%) | 1 (3.8%) | 6 (15.4%) | 1 (3.3%) | 13 (8.4%) |  |
| Agree | 18 (30.0%) | 11 (42.3%) | 15 (38.5%) | 14 (46.7%) | 58 (37.4%) |  |
| Strongly Agree | 33 (55.0%) | 13 (50.0%) | 16 (41.0%) | 15 (50.0%) | 77 (49.7%) |  |
